# The Dynamics of Functional Brain Networks Associated With Depressive Symptoms in a Nonclinical Sample

**DOI:** 10.3389/fncir.2020.570583

**Published:** 2020-09-18

**Authors:** Sonsoles Alonso Martínez, Gustavo Deco, Gert J. Ter Horst, Joana Cabral

**Affiliations:** ^1^Cognitive Neuroscience Center, Department of Biomedical Sciences of Cells and Systems, University Medical Center Groningen, Groningen, Netherlands; ^2^Center for Brain and Cognition, Computational Neuroscience Group, Department of Information and Communication Technologies, Universitat Pompeu Fabra, Barcelona, Spain; ^3^Institució Catalana de Recerca i Estudis Avançats (ICREA), Barcelona, Spain; ^4^Department of Psychiatry, University of Oxford, Oxford, United Kingdom; ^5^Center for Music in the Brain, Aarhus University, Aarhus, Denmark; ^6^Life and Health Sciences Research Institute (ICVS), University of Minho, Minho, Portugal

**Keywords:** depressive symptoms, dynamic FC, functional brain networks, nonclinical sample, resting-state fMRI, static FC, whole-brain

## Abstract

Brain function depends on the flexible and dynamic coordination of functional subsystems within distributed neural networks operating on multiple scales. Recent progress has been made in the characterization of functional connectivity (FC) at the whole-brain scale from a dynamic, rather than static, perspective, but its validity for cognitive sciences remains under debate. Here, we analyzed brain activity recorded with functional Magnetic Resonance Imaging from 71 healthy participants evaluated for depressive symptoms after a relationship breakup based on the conventional Major Depression Inventory (MDI). We compared both static and dynamic FC patterns between participants reporting high and low depressive symptoms. Between-group differences in static FC were estimated using a standard pipeline for network-based statistic (NBS). Additionally, FC was analyzed from a dynamic perspective by characterizing the occupancy, lifetime, and transition profiles of recurrent FC patterns. Recurrent FC patterns were defined by clustering the BOLD phase-locking patterns obtained using leading eigenvector dynamics analysis (LEiDA). NBS analysis revealed a brain subsystem exhibiting significantly lower within-subsystem correlation values in more depressed participants (high MDI). This subsystem predominantly comprised connections between regions of the default mode network (i.e., precuneus) and regions outside this network. On the other hand, LEiDA results showed that high MDI participants engaged more in a state connecting regions of the default mode, memory retrieval, and frontoparietal network (p-FDR = 0.012); and less in a state connecting mostly the visual and dorsal attention systems (p-FDR = 0.004). Although both our analyses on static and dynamic FC implicate the role of the precuneus in depressive symptoms, only including the temporal evolution of BOLD FC helped to disentangle over time the distinct configurations in which this region plays a role. This finding further indicates that a holistic understanding of brain function can only be gleaned if the temporal dynamics of FC is included.

## Introduction

The ability to flexibly engage in a variety of cognitive functions is crucial as it enables individuals to adapt to the constantly changing and sometimes threatening environment. Impairments in this ability might result in dysfunctional responses that ultimately contribute to psychopathology. For example, depressive symptoms in the general population are often triggered by the experience of stressful or upsetting life events (Kendler et al., 1999), such as the breakup of a relationship. Although not everyone responds to a breakup in the same manner, in some cases, individuals complain of grief, sadness, concentration difficulties, rumination thoughts, and lack of sleep (Field et al., 2009). This is of clinical relevance because the persistence of these symptoms over time places individuals at higher risk of developing a full-blown depressive episode (Cuijpers and Smit, 2004; Zisook et al., 2010; Karsten et al., 2011). While important progress has been made in elucidating the neural mechanisms involved in Major Depression Disorder (MDD), we still do not know how these mechanisms originate in the healthy brain. To address this question, we investigated the brain-behavior relationship in nonclinical individuals with varying degrees of depressive symptoms after a relationship breakup.

Accumulating evidence from neuroimaging experiments suggests that clinical and subclinical depressive symptoms may evolve due to abnormal interactions within and between several functional brain networks (for review, see Wang et al., 2012; Kaiser et al., 2015; Mulders et al., 2015; Helm et al., 2018). These include the default mode (DM) and dorsal attention (DAT) networks, supporting internally and externally oriented cognition, respectively, the ventral attention (VAT) network involved in salience detection; the frontoparietal (FP) and cingulo-opercular (CO) task-control networks, both regulating attention and emotion. Other studies applying whole-brain connectivity analyses have also found abnormalities in other less common networks in depression such as the sensorimotor (SMT) and visual (VIS) networks (Veer et al., 2010; Zeng et al., 2012; Yan et al., 2019).

Although abnormal functional connectivity (FC) has been largely documented in neuroimaging research of depression, these findings are sometimes in contradicting directions. Notably, most of these studies have examined brain FC from a static perspective, computing the correlation between BOLD signals over the entire recording time and extracting differences between condition using methods such as network-based statistic (NBS; Zalesky et al., 2010). Despite the advances, this approach fails to capture the alterations occurring in the temporal expression of known functional brain networks. Emerging data suggest that FC fluctuates over time (Chang and Glover, 2010; Hutchison et al., 2013; Calhoun et al., 2014; Preti et al., 2016) and that this dynamical property holds valuable information that is relevant for understanding the range of cognitive abilities that enable complex and adaptive behavior (Sakoğlu et al., 2010; Allen et al., 2014; Zalesky et al., 2014). Even in the absence of external stimulation, such as in resting-state, the brain is characterized by the constant exploration of time-varying patterns of coupling among brain regions (Deco et al., 2011; Hansen et al., 2015; Cabral et al., 2017a). In this study, we specifically measured brain activity while at rest because we hypothesize that signatures of a depressive mood may be intrinsically expressed in as changes in the dynamical behavior of specific functional networks. Indeed, a recent study on this same dataset demonstrated that increased depressive symptoms were associated with a reduced dynamic functional organization across the whole-brain and a more static regime over time (Alonso Martínez et al., 2020).

To date, several methods have been developed to investigate how these network configurations form, interact, and dissolve over time. Moreover, recent evidence suggests that the pattern of transitions between functional networks could serve a biomarker for brain disorders such as depression (Demirtaş et al., 2016; Kaiser et al., 2016; Wise et al., 2017; Figueroa et al., 2019). Although the most common method for evaluating dynamic FC has been the sliding window approach (Sakoğlu et al., 2010), other methods with higher temporal resolution have recently gained recognition because they reveal relevant and meaningful results. These include co-activation pattern analysis (Tagliazucchi et al., 2012; Karahanoğlu and Van De Ville, 2015) and phase-coherence pattern analysis (Glerean et al., 2012; Hellyer et al., 2015; Cabral et al., 2017b). While the former considers the frames from which regional activity exceeds a given threshold, the latter is sensitive to phase-locked synchronization of BOLD signal fluctuations. In particular, a method relying on the detection of recurrent BOLD phase-locking (PL) patterns, termed Leading Eigenvector Dynamics Analysis (LEiDA; Cabral et al., 2017b) has revealed subsystems that closely overlap with functional networks reported in the literature, such as the DM, FP, VAT, DAT, SMT, and VIS (Lord et al., 2019; Vohryzek et al., 2020). Notably, the dynamical properties of some of these networks (such as the probability of occurrence, duration, and transition probabilities) have been found to relate with cognitive performance (Cabral et al., 2017b), depressive history (Figueroa et al., 2019), the effects of psychoactive drugs (Lord et al., 2019), and even with the scores of an emotional reward task (Stark et al., 2019).

Based on the amount of literature that points out to disruption in functional connectivity, we employed LEiDA to characterize the dynamics of recurrent BOLD PL patterns (PL states) and quantify between-group differences in terms of the percentage of occupancy and lifetime of each PL state. Due to the non-stationary nature of resting-state FC, we predict that investigating the temporal dynamics of resting-state FC would be more advantageous in providing new information rather than assuming a static perspective on FC. To this end, we compared our results from the LEiDA approach to those obtained from the analysis of static FC using the NBS technique.

## Materials and Methods

### Participants

We analyzed a data set from a previously published behavioral study on the degree of depressive symptoms after a relationship breakup (Verhallen et al., 2019). This data set consists of 71 volunteers (38 women; age range, 18–25) who were in a relationship for at least 6 months, which ended in the preceding 6 months. Inclusion criteria included Western background, no history of neurological or psychiatric disorders, heterosexuality, and oral contraceptive use in women. The study protocol was approved by the Ethics Committee at the University Medical Center Groningen. Participants provided written informed consent before the study procedure.

Two of the 71 enrolled participants, were excluded after neuroimaging data preprocessing (see section “Image Preprocessing”), resulting in a total of 69 participants (between 18 and 25 years old, 37 women) for functional Magnetic Resonance Imaging (fMRI) data analysis.

#### Depressive Symptoms

The prevalence of depressive symptoms was measured using the Major Depression Inventory (MDI), which is based on the DSM-IV and the ICD-10 symptoms of depression (Bech et al., 2001; Olsen et al., 2003). The MDI is a brief self-report mood questionnaire, considered a reliable instrument for assessing depression symptoms in the general population (Cuijpers et al., 2007). It consists of 12 questions about the frequency of depressive symptoms experienced over the last 14 days. MDI scores can range from 0 to 50, with higher scores indicating higher levels of depressive symptoms. MDI scores varied from 1 to 45 with an average score of 14.3 (IQR = 7–21). Participants were split into 2 groups based on the conventional MDI cut-off point of 20 for depression. Nine-teen participants (16 women) scored above this threshold and were classified as “high MDI” (median MDI score = 29); 50 participants (21 women) had a score equal to or below this threshold and were classified as “low MDI” (median MDI score = 7). Supplementary Table S1 displays the characteristics of the study sample.

### Image Acquisition

The MRI session was conducted on a 3 T Philips Intera MRI scanner (Philips Medical Systems, Best, Netherlands) using a 32-channel SENSE head coil. T2^∗^-weighted images were obtained using Fast Field Echo Planar Imaging (EPI) pulse sequence with gap = 0.3 mm; slice thickness = 3.5 mm; TR = 2000 ms; TE = 30 ms; FoV in mm (RL × AP × FH) = 220 × 121.8 × 220; voxel size in mm = 3.44 × 3.44 × 3.30; flip angle = 70°; oriented parallel to the AC-PC transverse plane and recorded in descending order. For each participant, a total of 150 volumes (37 slices per volume) were collected in a 306-second scanning session. Participants used foam pads to reduce head motion and earplugs to minimize the noise of the scanner. They were instructed to close their eyes, move as little as possible, and let their mind flow without falling asleep.

### Image Analysis

Neuroimaging data analysis was performed using SPM12^1^ and in-house Matlab scripts using Matlab 2015b (The Mathworks Inc., Natick, MA, United States).

#### Image Preprocessing

The EPI images were spatially realigned to the first volume using rigid body transformations, and the mean EPI generated in this step was coregistered to the structural T1 image. The coregistered images were spatially normalized to MNI T1-template and resampled to 2 × 2 × 2 mm voxel size. The bounding box was changed to −90:90, −126:90, −72:108 to ensure overlap of all regions of interest (ROIs) with the bounding box for time series extraction. Then, eight nuisance variables (i.e., six head motion parameters, white matter signal, and cerebrospinal fluid signal) and their first-order temporal derivatives were regressed out. Subsequently, images were spatially smoothed using a Gaussian kernel of 8 mm full-width at half maximum. After preprocessing, two of the 71 participants were excluded: one due to neuroimaging data quality issues, and another due to excessive head motion (> 0.3 mm in translation, or >3 degree in rotation).

#### ROI Definition and BOLD Time Series Extraction

We used a fine-grained parcellation scheme consisting of 270 non-overlapping regions (5-mm-radio spheres). These regions were defined from the 264-region parcellation of the cortex proposed by Power et al. (2011) and adding 6 subcortical structures (bilateral amygdala, hippocampus and caudate) from the Harvard-Oxford Subcortical Structural Atlas, given their relevance in depression-related research. A group whole-brain mask was generated in MNI space to localize the parts of the brain that were free from susceptibility artifacts in all participants. Individual participant’s whole-brain masks were then evaluated to ensure that each region had more than 50% overlap with the group mask (i.e., common space for all participants), at 90% mean signal intensity. This step led to the exclusion of 47 ROIs from the Power et al. (2011) parcellation. For the remaining 223 regions, the BOLD time series were extracted as the average signal across voxels within each region (Supplementary Table S2).

#### Band-Pass Filtering of BOLD Time Series

The participant-specific set of 223 ROI-time series was bandpass filtered between 0.01 and 0.1 Hz, using a 9th order Butterworth filter, to discard low-frequency drifts (<0.01 Hz) and high frequency components associated to cardiac and respiratory signals (> 0.1 Hz).

### Static FC Analysis

Between-group differences in static FC were estimated with the NBS approach, implemented in the NBS Toolbox for Matlab R2015b. The NBS analysis, described by Zalesky et al. (2010), is a non-parametric technique that controls for the family-wise error rate (FWER), when mass-univariate testing is performed at every connection comprising the network, potentially offering a substantial gain in statistical power. Before using NBS, we calculated for each participant, the pairwise Pearson correlation coefficients between the filtered time series of each region. The calculated coefficients were stored in a 223 × 223 symmetric FC matrix (the 223 diagonal elements were removed). Following the NBS procedure, a *t*-test contrasting the two groups (low vs. high-MDI) was calculated for each pairwise correlation/connection. We controlled for the effect of gender given that there was a significant difference in the proportion of women and men in the 2 groups, *X*^2^(1, *N* = 69) = 9.9, *p* = 0.02 (Supplementary Table S1). Then, we defined a set of suprathreshold connections by identifying all links with a T > 3.5. Note that although the network threshold influences the extent of the returned network, the selection of this value is arbitrary. As recommended by Zalesky et al. (2010), we compared the returned network to those obtained with a less (T > 3.1) and a more (T > 4) restricted threshold. Links with positive and negative t-scores were calculated separately to identify connected components where participants with high MDI had either significantly higher or lower connectivity strength compared to participants with low MDI. We used 10,000 permutations to determine the significance of the network at alpha = 0.05 and using FWER to correct for multiple comparisons.

### Dynamic FC Analysis

The analysis of dynamic FC involved the characterization of recurrent BOLD PL patterns by applying LEiDA. This is a data-driven approach that relies on the leading eigenvector of the BOLD PL matrix at each single TR (Cabral et al., 2017b). Matlab scripts to performed LEiDA are publicly available at github.com/sonsolesalonsomartinez/LEiDA.

#### Dynamic BOLD PL Matrix

**FIGURE 1:**
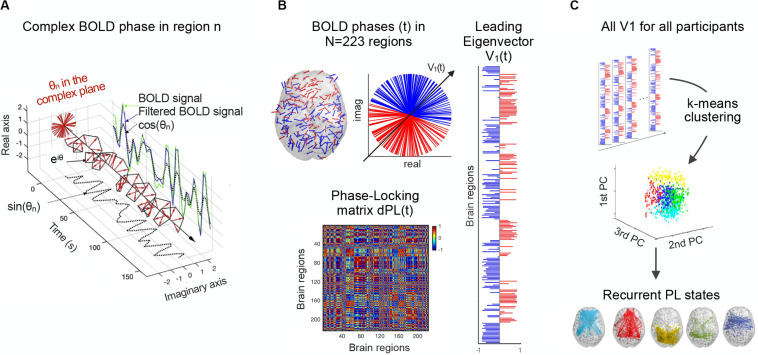
Detection of recurrent BOLD PL patterns. **(A)** For a given region, the BOLD signal (green) is first band-pass filtered between 0.01 and 0.1 Hz (blue) and then Hilbert transformed into an analytic signal, whose phase can be represented over time by e^iθ^ (black line) and at each TR (red arrows). **(B)** At a single time point, BOLD phases in all *N* = 223 regions can be represented in cortical space (left) and the complex plane (middle). The dPL(t) matrix captures the phase alignment between each pair of regions (bottom). The leading eigenvector of the dPL(t) matrix, V(t), is the vector that best captures the main orientation of all phases, where each element in V(t) corresponds to the projection of the phase of each region into V(t) (right). **(C)** All the leading eigenvectors V(t) are concatenated over participants and fed into a k-means clustering algorithm, which divides the pool of data points into a predefined number of clusters k. Each cluster centroid represents a recurrent PL state. dPL, dynamic phase-locking.

First, a whole-brain pattern of BOLD PL was obtained at each time point by computing the dynamic phase-locking matrix (dPL). The dPL estimates the phase alignment between each pair of brain regions, the value of which varies from 1 to −1, for signals changing in the same or opposite direction, respectively. In more detail, for each participant, the 223 bandpass filtered time series were first demeaned and Hilbert transformed to estimate the analytic phase of the averaged BOLD signals. The Hilbert transform expresses any given signal in polar coordinates, x(t) = A(t)cos(θ(t)), where A(t) is the instantaneous amplitude or the envelope, and θ(t), the instantaneous phase, or phase angle. As shown in Figure 1A, the cosine of the phase angle still captures the fluctuations of the BOLD signal. Given the phases of the BOLD signals, the phase alignment, dPL (n,p,t), for each pair of regions, n and p, at time t, is calculated using the cosine function, as in the following equation:

d⁢P⁢L⁢(n,p,t)=cos⁡(θ⁢(n,t)-θ⁢(p,t))

*dPL* is a tensor of size N × N × T, where *N* = 223 is the number of brain regions, and *T* = 148 is the number of recording frames in each scan (after removing the first and last volumes of the total 150 to account for the boundary distortions associated to the Hilbert transform). Two regions n and p with temporarily aligned BOLD signals (i.e., with similar angles) at a given TR will have a PL value close to 1 (cos (0°) = 1), while regions with orthogonally developing BOLD signals (e.g., one increasing at 45° and the other decreasing at 45°) will have zero PL value (i.e., cos (90°) = 0).

#### Leading Eigenvector of the PL Matrix

The second step is to calculate the leading eigenvector for the resulting dPL matrix (Figure 1B). For each dPL(t), the leading eigenvector V_1_(t), of dimension N × 1, captures the main orientation of BOLD phases over all regions. Therefore, instead of considering all (upper triangular) elements of the N × N dPL(t), the LEiDA approach considers only the eigenvector associated with the largest magnitude eigenvalue. This strategy substantially reduces the dimensionality of the data, from N(N−1)/2 to N. The sign (positive or negative) of the eigenvector elements can be used to separate brain regions in one of the two communities (blue or red) according to their BOLD−phase relationship (Newman, 2006). Elements of different signs indicate BOLD signals following different directions with respect to their projection onto the leading eigenvector. Because V and −V represent the same eigenvector, only the relative sign between regions is relevant. A convention was used ensuring that most of the elements have negative values. The magnitude of the eigenvector elements indicates the strength with which brain regions belong to the communities in which they are placed (Newman, 2006).

#### Detection of Recurrent BOLD PL States

The next step is to define recurrent BOLD PL states by applying a k-means clustering algorithm that divides the set of leading eigenvectors into a predefined number of clusters k (Figure 1C). Determining the optimal number of clusters is indeed a fundamental issue in partitioning a system and the most common solutions include methods for optimizing a criterion, such as elbow and silhouette. Here, we do not aim to detect the optimal number of clusters describing the entire resting-state dataset, but rather identify which clusters of PL states significantly (false discovery rate (FDR) adjusted *p*-value < 0.05) and consistently differ between the two groups in terms of occupancy or lifetime across different k-clustering solutions (these measures are described in section “Occupancy, Lifetime and Transition Probability Profiles of PL States”). To this end, a *k*-means clustering algorithm was run for 11 partition models by varying the number of clusters k from 4 to 14, with higher k resulting in more fine-grained configurations. Specifically, for each partition model, clustering the 10,212 leading eigenvectors, V_1_, (resulting from 69 participants and 148 TRs each) returns *k* N × 1 cluster centroids, Vc, each representing a recurrent state of BOLD PL. The brain subsystem related to each cluster centroid, Vc, can be represented as a network in cortical space, by (i) drawing each element as a sphere using the coordinates of the corresponding brain region; and (ii) using the value of Vc(n) to plot links between regions with positive sign, to highlight the network formed by the smallest community of synchronized brain regions. The fact that the smallest synchronized community in Vc represents the most meaningful brain subsystem has been demonstrated empirically in previous works (Lord et al., 2019; Larabi et al., 2020; Vohryzek et al., 2020), by revealing a highly significant overlap with functional systems from the literature (Yeo et al., 2011). However, these studies have considered a different parcellation atlas, consisting of 90 anatomically defined brain regions, and it has not been verified if it holds for a different and finer-grained parcellation scheme as we use here.

#### Functional Networks Assigned to Each PL State

To facilitate interpretation of BOLD PL states we used as a reference the consensus community assignments from Power et al. (2011) whereby each brain region is ascribed to one of 13 predefined functional networks (Supplementary Table S2). In addition, to allow for comparison with other studies, we also compared the resulting identified BOLD PL states to the seven resting-state networks (RSNs) from Yeo et al. (2011), following the methodology described in Vohryzek et al. (2020). However, to verify whether the smallest community still reveals the most functionally relevant brain subsystem in this work, the overlap was computed with both communities, considering either the positive (smallest community) or the negative (largest community) elements in Vc.

#### Occupancy, Lifetime and Transition Probability Profiles of PL States

The last step consists of the characterization of PL states in terms of (i) percentage of occupancy, calculated as the temporal percentage of epochs assigned to a given cluster centroid Vc, that is, the number of time points in which a PL state is active during the scan, divided by the total number of time points in a scan and multiplied by 100; (ii) lifetime, or duration, calculated as the mean number of consecutive epochs in the same state; and (iii) transition probability profiles, computed as the probability of switching from each PL state to any other PL state. Between-group differences in these measures were statistically assessed using a non-parametric permutation-based *t*-test with 10,000 gender-restricted permutations. We used gender-restricted permutations to account for the significant difference in the proportion of women and men in the two groups, *X*^2^(1, *N* = 69) = 9.9, *p* = 0.02 (see Supplementary Table S1). For each of the 11 partition models (from *k* = 4 to *k* = 14), multiple testing correction was conducted via FDR estimation at alpha = 0.05.

## Results

### Between-Group Differences in Static FC

**FIGURE 2:**
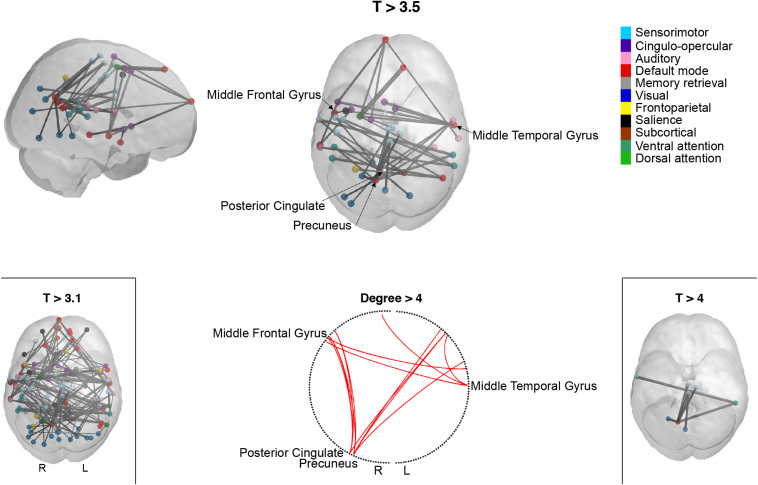
Participants with more depressive symptoms exhibit lower correlations within a specific subsystem revealed by network-based statistic (NBS). Cortical representation of the NBS-derived whole-brain functional network comprising reduced connectivity connections in high compared to low MDI for three different t-thresholds (T): T > 3.1, T > 3.5, and T > 4. The circle plot shows the highest degree (>4) nodes in the network. Spheres represent the coordinates of the NBS-derived regions. Spheres are color-coded according to the functional network they belong to, as described in Power et al. (2011). L, left; MDI, Major Depression Inventory; R, right.

Network-based statistic identified a single subsystem exhibiting significantly lower within-subsystem correlation values in high compared to low MDI. This consisted of 42 links between 39 brain regions (*T* = 3.5; p-FWER = 0.010). As shown in Figure 2 and Supplementary Table S3, these regions are distributed across the frontal, temporal, occipital, and parietal lobe. 81% of the total 42 edges connected regions of the DM network with regions from the VIS, AUD, SMT, CO, DAT, and VAT networks. Other connections included links between regions of the SMT network with regions of the VAT and the VIS network. The regions that exhibited the highest degree centrality were found within the DM network. Specifically, links with the right precuneus accounted for 9 of the 42 significant connections, followed by the right posterior cingulate, with seven connections. Since no standard threshold has been established, experimenting with a range of thresholds is recommended (Zalesky et al., 2010). We found that a lower threshold, *T* = 3.1, resulted in a large subsystem which could contain spurious weak connections. On the contrary, a higher threshold, *T* = 4, helped to emphasize the strongest connections. These consisted of seven links, five of which were connections between the precuneus and other regions within the DM network. No connected components showed significantly increased connectivity in high compared to low MDI for any of these thresholds.

### Between-Group Differences in Dynamic FC

#### Consistency of Between-Group Differences Across Partition Models

**FIGURE 3:**
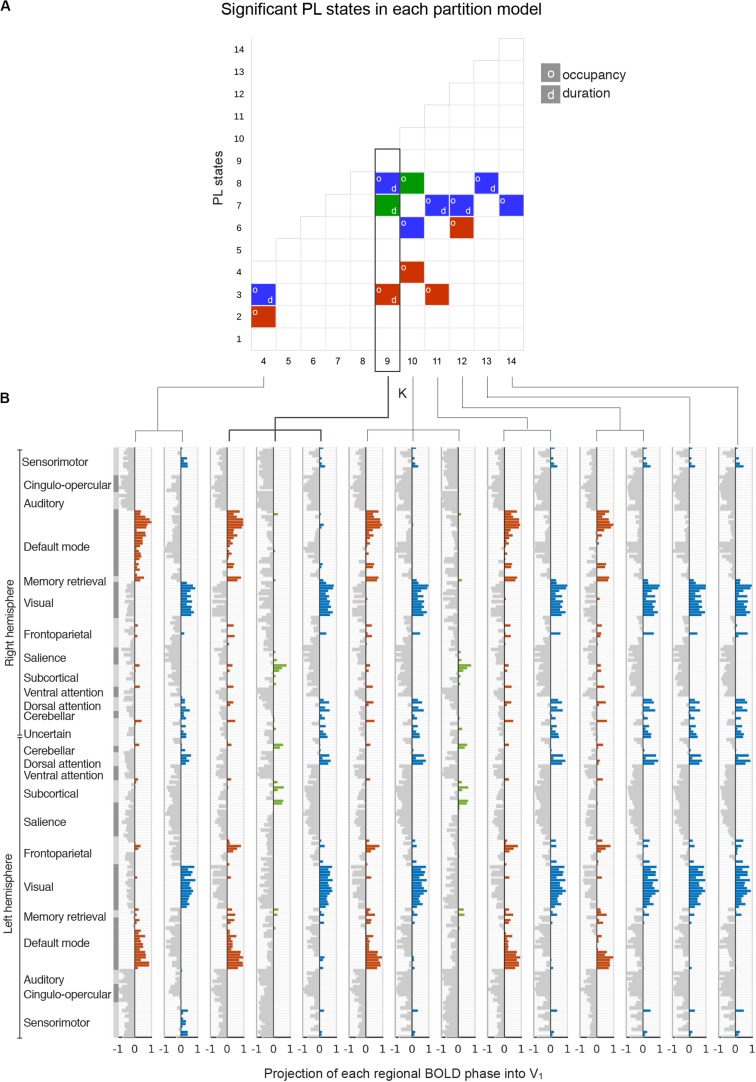
Detection of significant between-group differences in PL states across partition models. **(A)** Each square in the matrix represents a PL state (or cluster) obtained for each k-means partition model with k varying between *k* = 4 and *k* = 14 (*x*-axis). PL states that are significantly different between low and high MDI (p-FDR < 0.05), either in terms of percentage of occupancy (o) or duration (d), are indicated by colored squares. Since significantly different PL states obtained for different k were found to represent variant forms of 3 underlying configurations of BOLD phase locking (see panel B), we used a color-code (blue, red, green) to illustrate how these different configurations occurred across k. **(B)** The cluster centroids, Vc, of significantly different PL states identified in panel A are represented as bar plots. Here, each horizontal bar represents the BOLD phase in each of the *N* = 223 brain regions projected into the corresponding leading eigenvector (captured by the N elements in Vc). The regions n whose BOLD signal is phase-shifted (Vc(n) > 0) were colored using the same color-code from panel A. MDI, Major Depression Inventory; PL, phase-locking.

We first searched for the PL states that most significantly and consistently differentiated participants with high MDI from participants with low MDI. Across the 11 partition models explored, a total of 14 PL states were found to be significantly (p-FDR < 0.05) different between the two groups in terms of either percentage of occupancy or duration. Note that the multiple hypotheses being tested across partition models are not independent of each other, and therefore *p*-values were adjusted in each partition model separately. Figure 3A shows for each partition model (from *k* = 4 to *k* = 14 in the *x*-axis) the clusters that significantly differentiated participants with low and high MDI (in the *y*-axis clusters are sorted according to their percentage of occupancy). The clusters with the same color indicate that they refer to variant forms of the same underlying PL state, as can be observed in the vector format representation of these PL state centroids, Vc (Figure 3B). We classified the significant PL state centroids in three groups: the one highlighted in orange changed significantly between groups in 5 k-means solutions, the network highlighted in blue was significant for seven solutions and another PL state (green) showed significant between-group differences in two solutions. Since this last PL state was not consistent across cluster solutions, we did not consider it as a robust finding. For the subsequent analysis, we focused on the two PL states that significantly differed for high compared to low MDI consistently across five clustering solutions (i.e., *k* = 4, 9, 10, 11, and 12).

#### Relevant PL States

**FIGURE 4:**
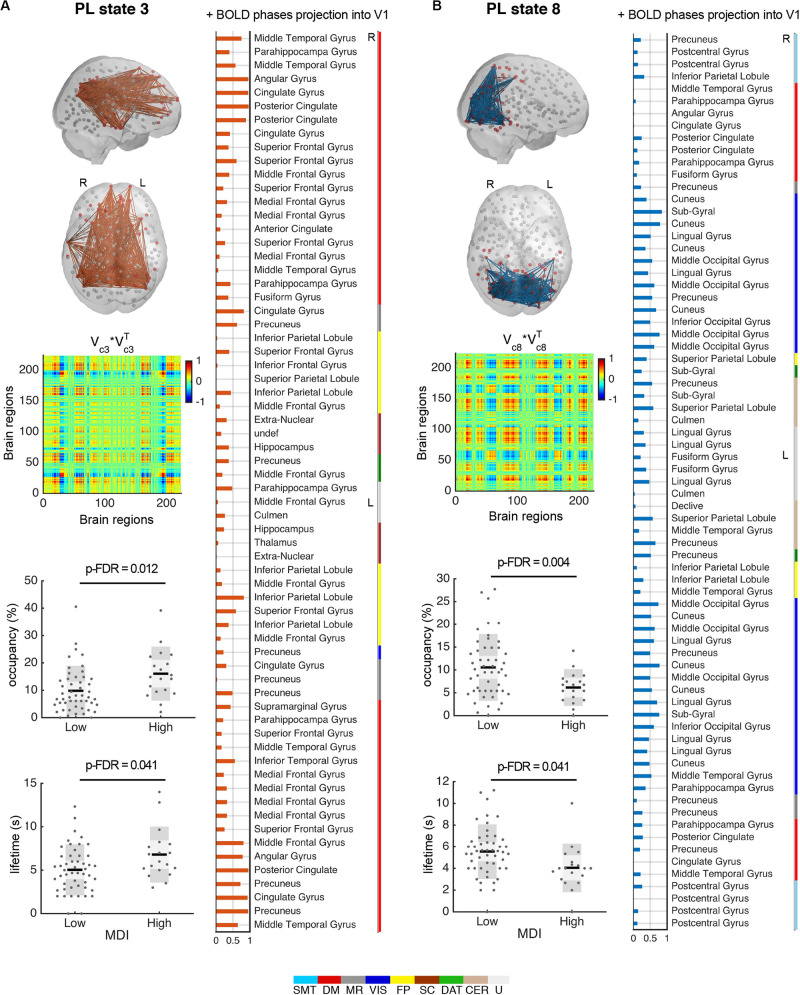
PL states 3 **(A)** and 8 **(B)** significantly differ for high compared to low MDI. (top left) Each PL state is represented in the cortical space, where functionally connected brain regions (represented as spheres) are colored in red. Links between vector elements with Vc(n) > 0.3 are drawn to highlight the configuration captured by each centroid. PL states are also represented as the outer product of Vc, which is a 223 × 223 matrix representing the number of brain regions, where positive (red) values indicate the product of Vc elements with the same sign, be they positive or negative. (right) Bar plot showing the elements in Vc with positive BOLD phase, representing the projection into the leading eigenvector. Vertical color bars indicate the network assigned to a given region. (bottom left) Significant (p-FDR < 0.05) differences in the percentage of occurrence and lifetime between low and high MDI. Dots represent individual data points; dark gray bars represent standard deviation, and lighter bars indicate standard error of the mean (represented by a horizontal line). Analysis via non-parametric permutation-based *t*-test (*N* = 69 participants). DAT, dorsal attention; DM, default mode; FP, frontoparietal task control; L, left; MDI, Major Depression Inventory; MR, memory retrieval; PL, phase-locking; R, right; SC, subcortical; SMT, sensorimotor; s, seconds; VIS, visual; CER, cerebellar; U, uncertain (visual-related network).

As described in the method section, we did not aim to determine the optimal number of clusters, but instead to identify the partition model that was able to capture the states that consistently and significantly distinguished participants with high vs. low MDI. We chose the partition model *k* = 9 because it captured the most significant changes between high and low MDI in two different BOLD PL states in terms of both their percentage of occupancy and duration. In particular, for the selected partition model (*k* = 9), PL state 3 (Figure 4A) occurred more in high MDI (16.1 ± 8.8%) compared to low MDI (9.8 ± 8%; p-FDR = 0.012, Hedges’ *g* = 0.77, medium to large effect size) with each occurrence lasting on average 6.8 ± 2.9 s compared to 5.0 ± 2.6 s in low MDI (p-FDR = 0.041, Hedges’ *g* = 0.67, medium to large effect size). The smallest community in this state mostly comprises regions of the DM, memory retrieval (MR), and FP networks as well as subcortical regions. Conversely, PL state 8 (Figure 4B) appeared less in high MDI (6.2 ± 3.4%) compared to (10.6 ± 6.6%) in low MDI (p-FDR = 0.004, Hedges’ *g* = 0.74, medium to large effect size) and lasted shorter (4.1 ± 2.0 s vs. 5.5 ± 2.3 s, p-FDR = 0.041, Hedges’ *g* = 0.63, medium to large effect size). This PL state comprised mostly connections of the VIS and DAT networks, but also the postcentral gyrus, angular gyrus, and regions of the so-called visual recognition network, namely the lingual and fusiform gyrus (Tao et al., 2013). Between-group differences for all PL states are provided in Supplementary Table S4.

#### The Repertoire of PL States Obtained With *k* = 9

**FIGURE 5:**
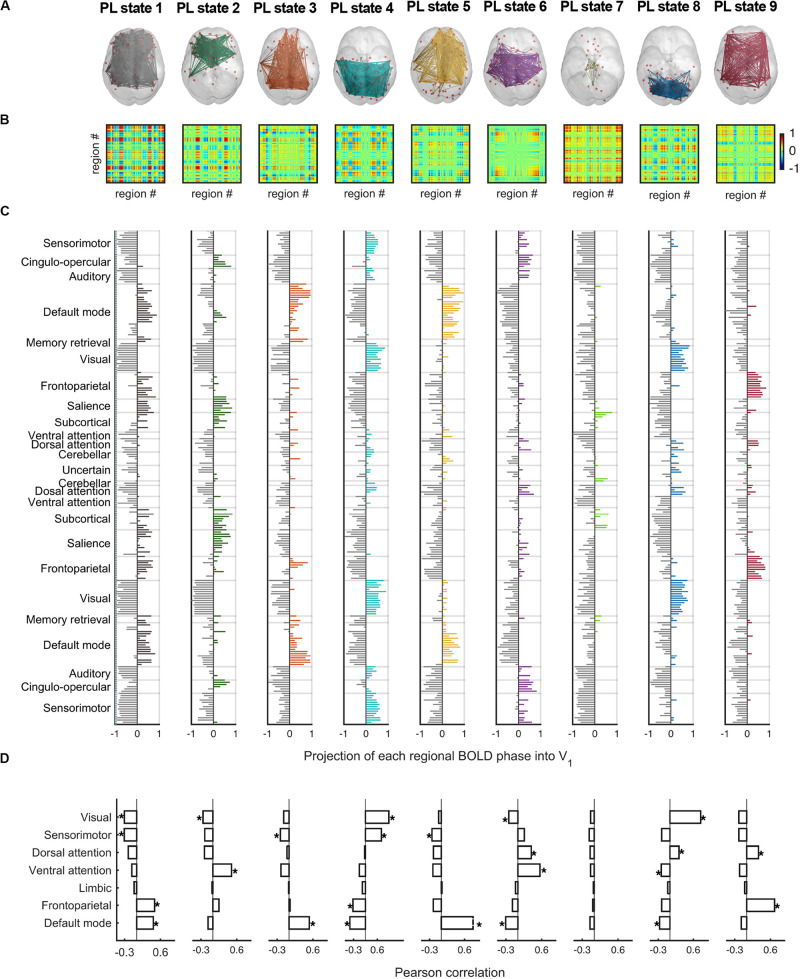
The repertoire of 9 PL states detected by clustering the set of leading eigenvectors into 9 clusters. **(A)** Cortical space representation of each PL state. Only functionally connected brain regions are drawn (red spheres). Links between vector elements with Vc(n) > 0.3 are displayed to highlight the configuration captured by each centroid. **(B)** Matrices (223 × 223) obtained calculating the outer product of Vc, where positive (red) values indicate the product of Vc elements with the same sign, be they positive or negative. **(C)** Bar plot showing the *N* = 223 elements in Vc, representing the projection of the BOLD phase in each region into the leading eigenvector. **(D)** Pearson correlation of the 9 PL states returned by our partition model (LEiDA for *k* = 9) and the seven resting-state networks defined in Yeo et al. (2011). *p-value < 0.001. PL, phase-locking.

Figure 5 displays the full repertoire of recurrent PL states obtained when partitioning the data into *k* = 9 clusters. These PL states are synchronization patterns that appear, dissolve, and reoccur over time in all participants during the entire resting-state fMRI recording session. PL states can be represented in cortical space (Figure 5A), in a matrix as the eigenvector’s outer product (Figure 5B) and vector format (Figure 5C). Because we used a fine-grained parcellation with 223 regions, we obtained a more detailed description of the implicated functional networks, revealing that each PL state involves functionally different sets of brain regions. In addition, to make it more comparable to the current literature, Figure 5D shows the spatial similarities between the PL states obtained here and the 7 RSNs defined in Yeo et al. (2011). From the Pearson’s correlation analysis, we found significantly (*p* < 0.001) positive correlations of state 1 (considering positive Vc elements) with the FP and the DM networks; state 2 with the VAT network; states 3 and 5 with the DM network; state 4 with the VIS and SMT networks; state 6 with the DAT and VAT networks; state 8 with the VIS and DAT networks; and state 9 with the FP and DAT networks. State 7, which does not overlap with any of the 7 RSNs proposed by Yeo, includes regions involved in executive control (mostly subcortical regions). Note that although PL states 3 and 5 both correlated exclusively with the DM network, these are two different states, with PL state 3 showing a weaker decoupling with the DAT and the FP network, compared to PL state 5.

#### The Role of the Precuneus From a Static to a Dynamic Perspective

**FIGURE 6:**
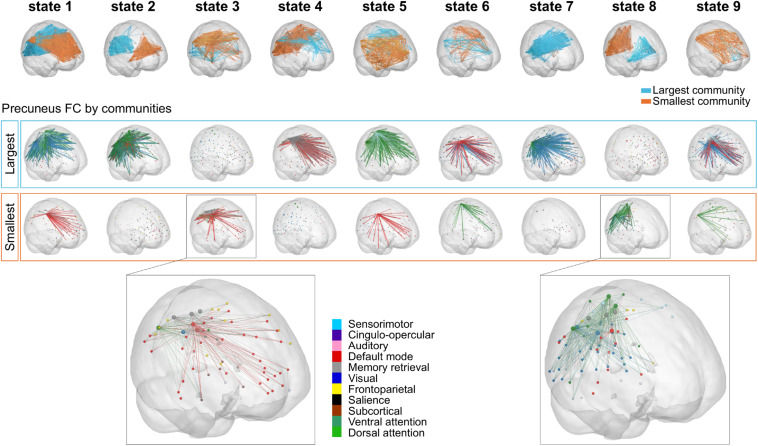
Precuneus FC for the repertoire of nine PL states. **(Top)** Subnetworks determined by the largest (blue) and smallest (orange) communities in each cluster centroids Vc. Note that Vc captures the main orientation of BOLD phases over all regions and that the sign (positive or negative) of the eigenvector elements are used to separate brain regions in one of the two communities (blue-largest or orange-smallest) according to their BOLD–phase relationship. Below each PL state, the cortical representation of the functional connections involving the precuneus regions for the largest **(Middle)** and smallest **(Bottom)** communities. Here, seeds were color-coded according to their assigned functional network, and links were color-coded according to the functional network each precuneus region belongs to. Note that PL states 3 and 8 (i.e., PL states that were expressed significantly different in high vs. low MDI) were zoomed in for visualization purposes (with larger spheres indicating regions of the precuneus). MDI, Major Depression Inventory; PL, phase-locking.

Considering the results obtained using NBS, where the precuneus was found to be the region exhibiting most dysconnectivity in high MDI, we investigated the specific role of the precuneus in the repertoire of recurring PL states obtained with *k* = 9 using LEiDA. While in Figures 5, the repertoire of BOLD PL states was represented by highlighting only the subset of brain regions belonging to the smallest community determined by the signs in Vc (following the previous literature), in Figure 6 (top) we consider the subnetworks determined by both the largest (blue) and smallest (orange) communities in each cluster centroids Vc. Below each PL state, we show for both communities, the connections involving the precuneus regions. Links were color-coded according to the functional network each precuneus region belongs to. All seeds were color-coded according to their assigned functional network. Noteworthily, in PL states 3 and 8 –the ones found to change most significantly between groups–, regions of the precuneus were found to be synchronized within the smallest community only. To facilitate visualization, these two states were zoomed in, with larger seeds indicating regions of the precuneus. We found that the parts of the precuneus involved in each PL state are functionally different and connect to regions within different functional networks. Specifically, while in PL state 3, the BOLD signal in the precuneus is synchronized mostly with regions of the DM network, in state 8, it is synchronized with regions of the VIS and DAT networks.

#### Transitions Between PL States

**FIGURE 7:**
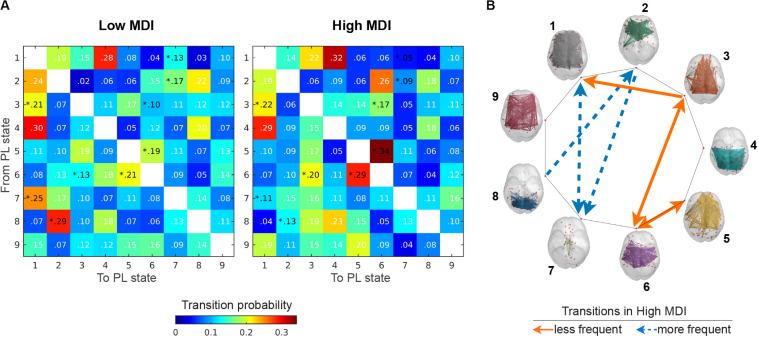
Transition probabilities between PL states. **(A)** Displays the switching matrices showing the probability of transition from a given PL state (rows) to any other PL states (columns) in low (left) and high MDI (right). The color bar is used to scale the transition probabilities between PL states. **(B)** Illustrates the transitions between PL states (rendered on the cortical surface) that significantly (*p* < 0.05) increased (solid orange line) and decreased (dashed blue line) for participants with high compared to low MDI. Analysis via a non-parametric permutation-based *t*-test. *p < 0.05. MDI, Major Depression Inventory; PL, phase-locking.

For the selected partition model *k* = 9, we also explored between-group differences with respect to the probability of transitioning from a given PL state to any other PL state. Figure 7A shows the matrices containing the mean transition probabilities for each group and Figure 7B illustrates the transitions that significantly (*p* < 0.05) increased (solid orange line) and decreased (dashed blue line) for participants with high, compared to low MDI. Overall, we found that for both groups, once in state 1 the most probable transition is to state 4 and from this state the most probable transition is back to state 1, forming a closed-loop between these two PL states. In high MDI, the second most probable transition is from state 5 to state 6, and once in state 6, back to state 5, forming another closed loop that appears less evident in low MDI. We also found an increase in the probability of transitioning from state 3 to state 1 and 6 and from state 6 back to state 3 in high MDI. Conversely, in low MDI, the second most probable transition was from state 8 to state 2, which together with the probability of transitioning from state 2 to 7, from state 7 to 1 and from state 1 back to state 7 was significantly lower in participants with high MDI.

### Assessing the Effect of Motion

We confirm that the results found in this study were not driven by differences in motion during the scanning session. We assessed motion by calculating the mean framewise displacement (FD) for each subject. FD measures movement of any given frame relative to the previous one. A *t*-test revealed that the mean FD was not significantly different for high (0.16 ± 0.04) compared to low MDI (0.15 ± 0.04; *t* = −1.42, *p* = 0.16). We also investigated the relationship between the expression of PL state 3 and 8 with the mean FD. No significant Pearson correlation was observed for state 3 in terms of percentage of occupancy (*r* = −0.12, *p* = 0.31) or lifetime (*r* = −0.05, *p* = 0.71). Nor there was a significant correlation of mean FD with the percentage of occupancy (*r* = 0.13, *p* = 0.31) or lifetime (*r* = 0.16, *p* = 0.18) of PL state 8.

## Discussion

We investigated potential differences in both static and dynamic patterns of BOLD FC for participants reporting high compared to low depressive symptoms after a relationship breakup. Even though both measures captured significant differences between the groups, our results demonstrate the advantage of capturing changes in the temporal expression of functional networks to gain novel insights into the psychophysiology of depressive symptomatology.

The application of the LEiDA approach revealed a repertoire of BOLD PL patterns that emerged, dissolved, and reoccurred over time in all participants, corroborating evidence for the dynamic nature of brain FC during rest. This result reinforces the conceptualization of dynamic FC as a multi-stable process where the connectivity patterns pass through multiple and reoccurring relatively stable states (Hutchison et al., 2013; Hansen et al., 2015; Vohryzek et al., 2020).

While the repertoire of PL states was similar across participants, the expression of two of these states was found to be significantly different between the two groups in terms of their percentage of occurrence and lifetime. In particular, there was an increased occupancy of a PL state characterized by DM network dominance in participants with higher depressive symptoms; and a decreased occupancy of a state overlapping with VIS and DAT networks. The significance of these two states was consistent across a range of partition models.

Both static and dynamic measures of FC implicate the role of the precuneus in depressive symptoms. However, including the temporal properties of phase coupling patterns helped to disentangle over time the distinct configurations in which the precuneus plays a role. Taken together, our findings using LEiDA emphasize the existence of BOLD PL patterns that are sensitive to distinguish participants with high versus low depressive symptoms even in a nonclinical sample.

### The Precuneus Accounts for the Overall Reduced Static FC in Participants With High MDI

By applying the NBS approach, we identified a single brain subsystem exhibiting significantly lower within-subsystem connectivity in participants with higher depressive symptoms. The links in this brain subsystem were mainly long-distance correlations between regions of the DM network and regions outside the DM network, with the right precuneus accounting for the largest number of them. The precuneus, as a core component of the DM network (Utevsky et al., 2014 but see also Margulies et al., 2009) may be important for self-referential processing (Fransson and Marrelec, 2008; Van Buuren et al., 2010) and has been commonly described with aberrant resting-state activity (Liu C.-H. et al., 2017; Li et al., 2018) and connectivity in patients with depression (Zhu et al., 2012; Dutta et al., 2019; Jacob et al., 2020). The study by Jacob et al. (2020) found reduced strength connectivity of this region to be linked to higher rumination tendency (i.e., an exaggerated focus on negative thoughts) in patients with depression. However, these maladaptive thinking patterns, often seen in patients with depression, have been associated with an increase, rather than a decrease in precuneus connectivity. Consequently, a sole consideration of the results obtained with static measures of FC would lead us to the supposition that there was a decreasing tendency in participants with high depressive symptoms to engage with processes concerning the self.

The precuneus, which is functionally divided into several regions, contributes to multiple networks and it is involved in a variety of cognitive processes including, but not limited to, visuospatial processing, episodic memory, and self-referential processes. In this context, investigating the dynamic configurations connecting the precuneus with other regions cannot be captured from a static perspective of FC, such as in Jacob et al. (2020), which ignores changes that might occur in the temporal domain.

### Dynamical Properties of FC Disentangle the Distinct Role of the Precuneus Over Time

By including the temporal properties of BOLD phase coupling we show that participants who reported more frequent depressive symptoms spend more time in a state (PL state 3) characterized by higher dominance of the DM network. This finding was previously interpreted as a sign of excessive self-referential and ruminative thoughts (Broyd et al., 2009; Christoff et al., 2009; Hamilton et al., 2011; Marchetti et al., 2012; Marusak et al., 2017) and has been previously linked to depressive symptoms in the general population (Wei et al., 2014). Evidence from EEG studies also shows that this predominance might already exist at the preclinical stages of depression (Knyazev et al., 2016). Rumination is considered to implicate a variety of cognitive and affective processes such as attention, self-referential encoding, and recall of autobiographical memories (Cooney et al., 2010). This functional diversity may be manifested in the involvement of other regions outside the DM network, as observed in PL state 3. These regions were part of the DM but also, the MR and FP networks as well as the hippocampus (Huijbers et al., 2011), and were mostly driven by connections with the precuneus.

Although there was another state (PL state 5) with a strong synchronization between the precuneus and other regions in the DM network, no differences were observed in the occurrence of this state between low and high MDI. Compared to PL state 3, the DM network coupling in PL state 5 was accompanied by a stronger decoupling with the rest of the brain, which was mostly driven by connections with other parts of the precuneus. This indicates that between-group differences involving DM network connectivity exceed connections within this network. It further supports the hypothesis that a shift of the precuneus connectivity to other regions outside the DM network relates to atypical connectivity patterns in depression (Dutta et al., 2019). In the same direction, Marusak et al. (2017) suggested that high synchrony between the DM and the executive control network was associated with self-reflective thoughts whereas increased FC within the DM and decreased connectivity between this network and the executive control network was associated to positive thoughts. Our finding seems supportive of the concept that DM network-mediated self-reflection becomes maladaptive when other networks integrate with the DM network (Sheline et al., 2010; Hamilton et al., 2015).

Conversely, participants with high depressive symptoms spend less time in a state (PL state 8) characterized by a shift in precuneus coupling away from the DM network, connecting regions of the VIS and DAT networks. In healthy participants, these two networks are known to be highly synchronized at rest (Beckmann et al., 2005). Regions of the DAT are thought to be responsible for goal-directed top-down processing by modulating activity in visual regions (Corbetta and Shulman, 2002; Bressler et al., 2008). In our study, such goal-directed processing of visual information (provoked by internal stimulation) appears to be less common in the presence of depressive symptoms. This observation is in line with a study reporting weaker functional dominance of the DAT in association with greater sadness and subclinical depression (Petrican et al., 2015). Other connections in PL state 8 included the inferior and superior parietal lobule, which have been proposed to be the main components of the executive control network, participating in the regulation and interpretation of sensory information. A study in college students with nonclinical depression suggested that alterations in these regions might be associated with abnormal emotional control of visual information processing (Wei et al., 2014). Despite the scarce literature implicating abnormalities in the VIS network, several studies have shown that depression can profoundly modify the visual and visual-attentional systems (Veer et al., 2010; Sacchet et al., 2016; Chen et al., 2019; Moreno-Ortega et al., 2019). For example, Moreno-Ortega et al. (2019) showed that the reduced VIS network connectivity found in patients with depression reversed after successful electroconvulsive therapy. More comparable to our analysis, (Zhi et al., 2018) found that patients with depression also spent less time in states of high connectivity with the visual system.

Almost all the entire precuneus established connections during the occurrence of PL state 8. In healthy controls, precuneus connectivity with cortical visual regions, angular gyrus, and temporal cortex suggest the potential involvement of this region in visual imagery processes (Fletcher et al., 1995). Furthermore, imagery vividness has been related to several posterior cortical regions, the fusiform gyrus, posterior cingulate, and parahippocampal gyrus, similarly, to those found in PL state 8. Accordingly, this state may reflect processes related to participants planning the future and remembering the past during the stimulus-free recording session.

In sum, participants with high depressive symptoms engaged more in a state characterized by an increased coupling of the precuneus with regions in the DM network (PL state 3), and less in a state where the precuneus shifts away from connections in this network (PL state 8). We proposed that this imbalance might result in maladaptive cognitive styles that ultimately contribute to sad mood and depressive symptoms, which aligns with other studies implicating abnormal FC of the precuneus in patients with depression (Sheline et al., 2009; Li et al., 2013; Chen et al., 2018).

### Strengths and Limitations

Three aspects of our results require further consideration. The first one relates to the controversial step in the preprocessing of resting-state FC data, global signal regression (GSR) (Uddin, 2017). Although the removal of global sources of variance attenuates motion and respiratory-related sources of noise (Fox et al., 2009; Murphy and Fox, 2017), GSR may also eliminate important information about ongoing neural activity (Liu T. T. et al., 2017). A recent study has shown that the global signal contains a rich source of information associated with behavior and trait-level cognition (Li et al., 2019). We chose not to regress out the global signal, given the potential functional relevance of this signal and to keep our analyses comparable with the literature using LEiDA where global signal was preserved (Cabral et al., 2017b; Figueroa et al., 2019; Lord et al., 2019; Larabi et al., 2020; Vohryzek et al., 2020). Yet, it is worth noting that including or removing the global signal might provide complementary insights into the brain’s functional organization (Murphy and Fox, 2017). Second, contrary to other studies using LEiDA (Cabral et al., 2017b; Figueroa et al., 2019; Lord et al., 2019; Stark et al., 2019; Larabi et al., 2020), we did not find a PL state where all BOLD signals followed a single global mode, (i.e., all signals projecting toward the same direction into the leading eigenvector). A possible explanation of this outcome could relate to differences in fMRI data preprocessing steps, particularly those involving white matter and CSF signal regression, as well as the definition of cortical areas, which may include CSF signal and variability across subjects. Therefore, efforts should be made to understand the impact of these and other preprocessing steps in the computation of dynamic FC. Third, although participants were instructed to keep their eyes closed, eye movements were not tracked, and therefore we cannot rule out the possibility that participants opened their eyes at some points during the resting-state scanning session. Although we found significant differences in a state (PL state 8) connecting regions of the VIS with other networks, these results do not seem to be explained by spontaneous eye-apertures. If our results were driven by the effect of feedforward visual input, we would also expect between-group differences in the other visual-related state (PL state 4), comprised of connections involving the VIS and SSM networks. Supporting this argument, a recent study showed that eyes closed/eyes open conditions significantly affected the patterns of connectivity between the VIS and the SSM networks, where they were mostly anticorrelated in eyes open condition, and correlated in the case of eyes closed (Agcaoglu et al., 2019).

While data analysis approaches of functional brain networks in the context of depression have varied widely, most of them have assessed FC within and between specific networks or regions of interest, of which sensory networks have been considered less of an imperative. On the other hand, when whole-brain analyses are performed, observing abnormalities in the FC involving the visual network are not as rare (Veer et al., 2010; Zeng et al., 2012; Borchardt et al., 2015; Wu et al., 2016; Moreno-Ortega et al., 2019; Schultz et al., 2019; Yan et al., 2019; Liu et al., 2020). These and our results, emphasize the potential implications of this network in the development of the disorder. Last, using LEiDA we found patterns that were previously implicated in depressive symptoms, such as increased expression of a state characterized by DM network dominance in participants with high depressive symptoms. However, we did not find abnormalities in FC involving both the DM and FP networks together, in contrast with what has been frequently found in patients with depression (Kaiser et al., 2015; Figueroa et al., 2019). We propose that this discrepancy could be ascribed to differences in population samples. Unlike most studies on depression, our study investigated the level of current depressive symptoms in a nonclinical population. Therefore, the observed alterations in FC found here seem to be associated with the current experience of depressive symptoms, which may cease to exist as participants recover from the breakup.

## Conclusion

In this study, we demonstrate the potential of whole-brain analyses of dynamic FC for investigating the psychophysiology of depressive symptoms in nonclinical populations. We find that participants with higher depressive symptoms spend significantly more time in a brain activity pattern related to self-referential thoughts and significantly less time in a pattern related to thinking about the past and planning the future. Together, these changes in brain activity patterns may account for the rumination tendency observed in depressive disorders. These findings encourage further research on depressive symptoms in the general population, to develop strategies that prevent these symptoms to trigger a full depressive episode. More importantly, by comparing and evaluating the results obtained from dynamic versus static FC analysis, we were able to demonstrate that a holistic understanding of brain function can only be gleaned if the temporal dynamics of functional connectivity is included.

## Data Availability Statement

The datasets presented in this article are not readily available because participants have not given consent to have their data publicly stored. The vectors representing the cluster centroids of BOLD PL patterns and the corresponding cluster time courses for each participant are available for download at github.com/sonsolesalonsomartinez/LEiDA. Requests to access the datasets should be directed to GH, g.j.ter.horst@umcg.nl.

## Ethics Statement

The studies involving human participants were reviewed and approved by Ethics Committee at the University Medical Center Groningen. The patients/participants provided their written informed consent to participate in this study.

## Author Contributions

GD, JC, and SAM conceptualized the analysis. GH provided resources and obtained funding for this study. JC contributed with codes, supervised the analysis, and revised the manuscript. SAM conducted all data analyses and wrote the manuscript. All authors participated in the discussion of the ideas, read and approved the submitted version.

## Conflict of Interest

The authors declare that the research was conducted in the absence of any commercial or financial relationships that could be construed as a potential conflict of interest.

## Footnotes

1 http://www.fil.ion.ucl.ac.uk

## References

[B1] Agcaoglu O. Wilson T. W. Wang Y. Stephen J. Calhoun V. D. (2019). Resting state connectivity differences in eyes open versus eyes closed conditions. *Hum. Brain Mapp.* 40 2488–2498. 10.1002/hbm.24539 30720907 PMC6865559

[B2] Allen E. A. Damaraju E. Plis S. M. Erhardt E. B. Eichele T. Calhoun V. D. (2014). Tracking whole-brain connectivity dynamics in the resting state. *Cereb. Cortex* 24 663–676. 10.1093/cercor/bhs352 23146964 PMC3920766

[B3] Alonso Martínez S. Marsman J.-B. C. Kringelbach M. L. Deco G. ter Horst G. J. (2020). Reduced spatiotemporal brain dynamics are associated with increased depressive symptoms after a relationship breakup. *NeuroImage Clin.* 27:102299. 10.1016/j.nicl.2020.102299 32516738 PMC7284067

[B4] Bech P. Rasmussen N. A. Olsen L. R. Noerholm V. Abildgaard W. (2001). The sensitivity and specificity of the major depression inventory, using the present state examination as the index of diagnostic validity. *J. Affect. Disord.* 66 159–164. 10.1016/S0165-0327(00)00309-8 11578668

[B5] Beckmann C. F. DeLuca M. Devlin J. T. Smith S. M. (2005). Investigations into resting-state connectivity using independent component analysis. *Philos. Trans. R. Soc. B Biol. Sci.* 360 1001–1013. 10.1098/rstb.2005.1634 16087444 PMC1854918

[B6] Borchardt V. Krause A. L. Starck T. Nissilä J. Timonen M. Kiviniemi V. (2015). Graph theory reveals hyper-functionality in visual cortices of seasonal affective disorder patients. *World J. Biol. Psychiatry* 16 123–134. 10.3109/15622975.2014.966144 25363311

[B7] Bressler S. L. Tang W. Sylvester C. M. Shulman G. L. Corbetta M. (2008). Top-down control of human visual cortex by frontal and parietal cortex in anticipatory visual spatial attention. *J. Neurosci.* 28 10056–10061. 10.1523/JNEUROSCI.1776-08.2008 18829963 PMC2583122

[B8] Broyd S. J. Demanuele C. Debener S. Helps S. K. James C. J. Sonuga-Barke E. J. S. (2009). Default-mode brain dysfunction in mental disorders: a systematic review. *Neurosci. Biobehav. Rev.* 33 279–296. 10.1016/j.neubiorev.2008.09.002 18824195

[B9] Cabral J. Kringelbach M. L. Deco G. (2017a). Functional connectivity dynamically evolves on multiple time-scales over a static structural connectome: models and mechanisms. *Neuroimage* 160 84–96. 10.1016/j.neuroimage.2017.03.045 28343985

[B10] Cabral J. Vidaurre D. Marques P. Magalhães R. Silva Moreira P. Miguel Soares J. (2017b). Cognitive performance in healthy older adults relates to spontaneous switching between states of functional connectivity during rest. *Sci. Rep.* 7 1–13. 10.1038/s41598-017-05425-7 28698644 PMC5506029

[B11] Calhoun V. D. D. Miller R. Pearlson G. Adali T. Adalı T. (2014). The chronnectome: time-varying connectivity networks as the next frontier in fMRI data discovery. *Neuron* 84 262–274. 10.1016/j.neuron.2014.10.015 25374354 PMC4372723

[B12] Chang C. Glover G. H. (2010). Time-frequency dynamics of resting-state brain connectivity measured with fMRI. *Neuroimage* 50 81–98. 10.1016/j.neuroimage.2009.12.011 20006716 PMC2827259

[B13] Chen H. Liu K. Zhang B. Zhang J. Xue X. Lin Y. (2019). More optimal but less regulated dorsal and ventral visual networks in patients with major depressive disorder. *J. Psychiatr. Res.* 110 172–178. 10.1016/J.JPSYCHIRES.2019.01.005 30654314

[B14] Chen V. C. H. Liu Y. C. Chao S. H. McIntyre R. S. Cha D. S. Lee Y. (2018). Brain structural networks and connectomes: the brain–obesity interface and its impact on mental health. *Neuropsychiatr. Dis. Treat.* 14 3199–3208. 10.2147/NDT.S180569 30538478 PMC6263220

[B15] Christoff K. Gordon A. M. Smallwood J. Smith R. Schooler J. W. (2009). Experience sampling during fMRI reveals default network and executive system contributions to mind wandering. *Proc. Natl. Acad. Sci. U.S.A.* 106 8719–8724. 10.1073/pnas.0900234106 19433790 PMC2689035

[B16] Cooney R. E. Joormann J. Eugène F. Dennis E. L. Gotlib I. H. (2010). Neural correlates of rumination in depression. *Cogn. Affect. Behav. Neurosci.* 10 470–478. 10.3758/CABN.10.4.470 21098808 PMC4476645

[B17] Corbetta M. Shulman G. L. (2002). Control of goal-directed and stimulus-driven attention in the brain. *Nat. Rev. Neurosci.* 3 201–215. 10.1038/nrn755 11994752

[B18] Cuijpers P. Smit F. (2004). Subthreshold depression as a risk indicator for major depressive disorder: a systematic review of prospective studies. *Acta Psychiatr. Scand.* 109 325–331. 10.1111/j.1600-0447.2004.00301.x 15049768

[B19] Cuijpers P. van Straten A. Warmerdam L. (2007). Behavioral activation treatments of depression: a meta-analysis. *Clin. Psychol. Rev.* 27 318–326. 10.1016/j.cpr.2006.11.001 17184887

[B20] Deco G. Jirsa V. K. McIntosh A. R. (2011). Emerging concepts for the dynamical organization of resting-state activity in the brain. *Nat. Rev. Neurosci.* 12 43–56. 10.1038/nrn2961 21170073

[B21] Demirtaş M. Tornador C. Falcón C. López-Solà M. Hernández-Ribas R. Pujol J. (2016). Dynamic functional connectivity reveals altered variability in functional connectivity among patients with major depressive disorder. *Hum. Brain Mapp.* 37 2918–2930. 10.1002/hbm.23215 27120982 PMC5074271

[B22] Dutta A. McKie S. Downey D. Thomas E. Juhasz G. Arnone D. (2019). Regional default mode network connectivity in major depressive disorder: modulation by acute intravenous citalopram. *Transl. Psychiatry* 9 1–9. 10.1038/s41398-019-0447-0 30877271 PMC6420575

[B23] Field T. Diego M. Pelaez M. Deeds O. Delgado J. (2009). Breakup distress in university students. *Adolescence* 44 705–727. 20432597

[B24] Figueroa C. A. Cabral J. Mocking R. J. T. Rapuano K. M. van Hartevelt T. J. Deco G. (2019). Altered ability to access a clinically relevant control network in patients remitted from major depressive disorder. *Hum. Brain Mapp.* 40 2771–2786. 10.1002/hbm.24559 30864248 PMC6865599

[B25] Fletcher P. C. Frith C. D. Baker S. C. Shallice T. Frackowiak R. S. J. Dolan R. J. (1995). The mind’s eye—recuneus activation in memory-related imagery. *Neuroimage* 2 195–200. 10.1006/nimg.1995.1025 9343602

[B26] Fransson P. Marrelec G. (2008). The precuneus/posterior cingulate cortex plays a pivotal role in the default mode network: evidence from a partial correlation network analysis. *Neuroimage* 42 1178–1184. 10.1016/j.neuroimage.2008.05.059 18598773

[B27] Fox M. D. Zhang D. Snyder A. Z. Raichle M. E. (2009). The global signal and observed anticorrelated resting state brain networks. *J. Neurophysiol.* 101, 3270–3283. 10.1152/jn.90777.2008 19339462 PMC2694109

[B28] Glerean E. Salmi J. Lahnakoski J. M. Jääskeläinen I. P. Sams M. (2012). Functional magnetic resonance imaging phase synchronization as a measure of dynamic functional connectivity. *Brain Connect.* 2 91–101. 10.1089/brain.2011.0068 22559794 PMC3624768

[B29] Hamilton J. P. Farmer M. Fogelman P. Gotlib I. H. (2015). Depressive rumination, the default-mode network, and the dark matter of clinical neuroscience. *Biol. Psychiatry* 78 224–230. 10.1016/j.biopsych.2015.02.020 25861700 PMC4524294

[B30] Hamilton J. P. Furman D. J. Chang C. Thomason M. E. Dennis E. Gotlib I. H. (2011). Default-mode and task-positive network activity in major depressive disorder: implications for adaptive and maladaptive rumination. *August* 70 327–333. 10.1016/j.biopsych.2011.02.003 21459364 PMC3144981

[B31] Hansen E. C. A. Battaglia D. Spiegler A. Deco G. Jirsa V. K. (2015). Functional connectivity dynamics: modeling the switching behavior of the resting state. *Neuroimage* 105 525–535. 10.1016/j.neuroimage.2014.11.001 25462790

[B32] Hellyer P. J. Scott G. Shanahan M. Sharp D. J. Leech R. (2015). Cognitive flexibility through metastable neural dynamics is disrupted by damage to the structural connectome. *J. Neurosci.* 35 9050–9063. 10.1523/JNEUROSCI.4648-14.2015 26085630 PMC4469735

[B33] Helm K. Viol K. Weiger T. M. Tass P. A. Grefkes C. del Monte D. (2018). Neuronal connectivity in major depressive disorder: a systematic review. *Neuropsychiatr. Dis. Treat.* 14 2715–2737. 10.2147/NDT.S170989 30425491 PMC6200438

[B34] Huijbers W. Pennartz C. M. A. Cabeza R. Daselaar S. M. (2011). The hippocampus is coupled with the default network during memory retrieval but not during memory encoding. *PLoS One* 6:e0017463. 10.1371/journal.pone.0017463 21494597 PMC3073934

[B35] Hutchison R. M. Womelsdorf T. Allen E. A. Bandettini P. A. Calhoun V. D. Corbetta M. (2013). Dynamic functional connectivity: promise, issues, and interpretations. *Neuroimage* 80 5–79. 10.1016/j.neuroimage.2013.05.079 23707587 PMC3807588

[B36] Jacob Y. Morris L. S. Huang K. H. Schneider M. Rutter S. Verma G. (2020). Neural correlates of rumination in major depressive disorder: a brain network analysis. *NeuroImage Clin.* 25:102142. 10.1016/j.nicl.2019.102142 31901654 PMC6940660

[B37] Kaiser R. H. Andrews-Hanna J. R. Wager T. D. Pizzagalli D. A. (2015). Large-scale network dysfunction in major depressive disorder: a meta-analysis of resting-state functional connectivity. *JAMA Psychiatry* 72 603–611. 10.1001/jamapsychiatry.2015.0071 25785575 PMC4456260

[B38] Kaiser R. H. Whitfield-Gabrieli S. Dillon D. G. Goer F. Beltzer M. Minkel J. (2016). Dynamic resting-state functional connectivity in major depression. *Neuropsychopharmacology* 41 1822–1830. 10.1038/npp.2015.352 26632990 PMC4869051

[B39] Karahanoğlu F. I. Van De Ville D. (2015). Transient brain activity disentangles fMRI resting-state dynamics in terms of spatially and temporally overlapping networks. *Nat. Commun.* 6 1–10. 10.1038/ncomms8751 26178017 PMC4518303

[B40] Karsten J. Hartman C. A. Smit J. H. Zitman F. G. Beekman A. T. F. Cuijpers P. (2011). Psychiatric history and subthreshold symptoms as predictors of the occurrence of depressive or anxiety disorder within 2 years. *Br. J. Psychiatry* 198 206–212. 10.1192/bjp.bp.110.080572 21357879

[B41] Kendler K. S. Karkowski L. M. Prescott C. A. (1999). Causal relationship between stressful life events and the onset of major depression. *Am. J. Psychiatry* 156 837–841. 10.1176/ajp.156.6.837 10360120

[B42] Knyazev G. G. Savostyanov A. N. Bocharov A. V. Tamozhnikov S. S. Saprigyn A. E. (2016). Task-positive and task-negative networks and their relation to depression: EEG beamformer analysis. *Behav. Brain Res.* 306 160–169. 10.1016/J.BBR.2016.03.033 27001453

[B43] Larabi D. I. Renken R. J. Cabral J. Marsman J.-B. C. Aleman A. Ćurčić-Blake B. (2020). Trait self-reflectiveness relates to time-varying dynamics of resting state functional connectivity and underlying structural connectomes: role of the default mode network. *Neuroimage* 219:116896. 10.1016/j.neuroimage.2020.116896 32470573

[B44] Li B. Liu L. Friston K. J. Shen H. Wang L. Zeng L. L. (2013). A treatment-resistant default mode subnetwork in major depression. *Biol. Psychiatry* 74 48–54. 10.1016/j.biopsych.2012.11.007 23273724

[B45] Li G. Rossbach K. Zhang A. Liu P. Zhang K. (2018). Resting-state functional changes in the precuneus within first-episode drug-naive patients with MDD. *Neuropsychiatr. Dis. Treat.* 14 1991–1998. 10.2147/NDT.S168060 30122932 PMC6086096

[B46] Li J. Bolt T. Bzdok D. Nomi J. S. Yeo B. T. T. Spreng R. N. (2019). Topography and behavioral relevance of the global signal in the human brain. *Sci. Rep.* 9:14286. 10.1038/s41598-019-50750-8 31582792 PMC6776616

[B47] Liu C.-H. Ma X. Yuan Z. Song L.-P. Jing B. Lu H.-Y. (2017). Decreased resting-state activity in the precuneus is associated with depressive episodes in recurrent depression. *J. Clin. Psychiatry* 78 e372–e382. 10.4088/JCP.15m10022 28297595

[B48] Liu T. T. Nalci A. Falahpour M. (2017). The global signal in fMRI: nuisance or information? *Neuroimage* 150, 213–229. 10.1016/j.neuroimage.2017.02.036 28213118 PMC5406229

[B49] Liu Y. Chen Y. Liang X. Li D. Zheng Y. Zhang H. (2020). Altered resting-state functional connectivity of multiple networks and disrupted correlation with executive function in major depressive disorder. *Front. Neurol.* 11:272. 10.3389/fneur.2020.00272 32411071 PMC7198729

[B50] Lord L. D. Expert P. Atasoy S. Roseman L. Rapuano K. Lambiotte R. (2019). Dynamical exploration of the repertoire of brain networks at rest is modulated by psilocybin. *Neuroimage* 199 127–142. 10.1016/j.neuroimage.2019.05.060 31132450

[B51] Marchetti I. Koster E. H. W. Sonuga-Barke E. J. De Raedt R. (2012). The Default Mode Network and recurrent depression: a neurobiological model of cognitive risk factors. *Neuropsychol. Rev.* 22 229–251. 10.1007/s11065-012-9199-9 22569771

[B52] Margulies D. S. Vincent J. L. Kelly C. Lohmann G. Uddin L. Q. Biswal B. B. (2009). Precuneus shares intrinsic functional architecture in humans and monkeys. *Proc. Natl. Acad. Sci. U.S.A.* 106 20069–20074. 10.1073/pnas.0905314106 19903877 PMC2775700

[B53] Marusak H. A. Calhoun V. D. Brown S. Crespo L. M. Sala-Hamrick K. Gotlib I. H. (2017). Dynamic functional connectivity of neurocognitive networks in children. *Hum. Brain Mapp.* 38 97–108. 10.1002/hbm.23346 27534733 PMC5796541

[B54] Moreno-Ortega M. Prudic J. Rowny S. Patel G. H. Kangarlu A. Lee S. (2019). Resting state functional connectivity predictors of treatment response to electroconvulsive therapy in depression. *Sci. Rep.* 9:5071. 10.1038/s41598-019-41175-4 30911075 PMC6433903

[B55] Mulders P. C. van Eijndhoven P. F. Schene A. H. Beckmann C. F. Tendolkar I. (2015). Resting-state functional connectivity in major depressive disorder: a review. *Neurosci. Biobehav. Rev.* 56 330–344. 10.1016/j.neubiorev.2015.07.014 26234819

[B56] Murphy K. Fox M. D. (2017). Towards a consensus regarding global signal regression for resting state functional connectivity MRI. *Neuroimage* 154 169–173. 10.1016/j.neuroimage.2016.11.052 27888059 PMC5489207

[B57] Newman M. E. J. (2006). Finding community structure in networks using the eigenvectors of matrices. *Phys. Rev. E Stat. Nonlinear, Soft Matter Phys.* 74:036104. 10.1103/PhysRevE.74.036104 17025705

[B58] Olsen L. R. Jensen D. V. Noerholm V. Martiny K. Bech P. (2003). The internal and external validity of the major depression inventory in measuring severity of depressive states. *Psychol. Med.* 33 351–356. 10.1017/S0033291702006724 12622314

[B59] Petrican R. Saverino C. Shayna Rosenbaum R. Grady C. (2015). Inter-individual differences in the experience of negative emotion predict variations in functional brain architecture. *Neuroimage* 123 80–88. 10.1016/J.NEUROIMAGE.2015.08.031 26302674 PMC4898956

[B60] Power J. D. Cohen A. L. Nelson S. M. Wig G. S. Barnes K. A. Church J. A. (2011). Functional network organization of the human brain. *Neuron* 72 665–678. 10.1016/j.neuron.2011.09.006 22099467 PMC3222858

[B61] Preti M. G. Bolton T. A. Van De Ville D. De Ville D. V. (2016). The dynamic functional connectome: state-of-the-art and perspectives. *Neuroimage* 160 41–54. 10.1016/j.neuroimage.2016.12.061 28034766

[B62] Sacchet M. D. Ho T. C. Connolly C. G. Tymofiyeva O. LeWinn K. Z. Han L. K. M. (2016). Large-scale hypoconnectivity between resting-state functional networks in unmedicated adolescent major depressive disorder. *Neuropsychopharmacology* 41 2951–2960. 10.1038/npp.2016.76 27238621 PMC5061890

[B63] Sakoğlu Ü Pearlson G. D. Kiehl K. A. Wang Y. M. Michael A. M. Calhoun V. D. (2010). A method for evaluating dynamic functional network connectivity and task-modulation: application to schizophrenia. *Magn. Reson. Mater. Physics, Biol. Med.* 23 351–366. 10.1007/s10334-010-0197-8 20162320 PMC2891285

[B64] Schultz D. H. Ito T. Solomyak L. I. Chen R. H. Mill R. D. Anticevic A. (2019). Global connectivity of the fronto-parietal cognitive control network is related to depression symptoms in the general population. *Netw. Neurosci.* 3 107–123. 10.1162/netn_a_00056 30793076 PMC6326740

[B65] Sheline Y. I. Barch D. M. Price J. L. Rundle M. M. Vaishnavi S. N. Snyder A. Z. (2009). The default mode network and self-referential processes in depression. *Proc. Natl. Acad. Sci. U.S.A.* 106 1942–1947. 10.1073/pnas.0812686106 19171889 PMC2631078

[B66] Sheline Y. I. Price J. L. Yan Z. Mintun M. A. (2010). Resting-state functional MRI in depression unmasks increased connectivity between networks via the dorsal nexus. *Proc. Natl. Acad. Sci. U.S.A.* 107 11020–11025. 10.1073/pnas.1000446107 20534464 PMC2890754

[B67] Stark E. A. Cabral J. Riem M. M. E. Van IJzendoorn M. H. Stein A. Kringelbach M. L. (2019). The power of smiling: the adult brain networks underlying learned infant emotionality. *Cereb. Cortex* 30 2019–2029. 10.1093/cercor/bhz219 32129828 PMC7297298

[B68] Tagliazucchi E. Balenzuela P. Fraiman D. Chialvo D. R. (2012). Criticality in large-scale brain fMRI dynamics unveiled by a novel point process analysis. *Front. Physiol.* 3:15. 10.3389/fphys.2012.00015 22347863 PMC3274757

[B69] Tao H. Guo S. Ge T. Kendrick K. M. Xue Z. Liu Z. (2013). Depression uncouples brain hate circuit. *Mol. Psychiatry* 18 101–111. 10.1038/mp.2011.127 21968929 PMC3526729

[B70] Uddin L. Q. (2017). Mixed signals: on separating brain signal from noise. *Trends Cogn. Sci.* 21 405–406. 10.1016/j.tics.2017.04.002 28461113 PMC6033047

[B71] Utevsky A. V. Smith D. V. Huettel S. A. (2014). Precuneus is a functional core of the default-mode network. *J. Neurosci.* 34 932–940. 10.1523/JNEUROSCI.4227-13.2014 24431451 PMC3891968

[B72] Van Buuren M. Gladwin T. E. Zandbelt B. B. Kahn R. S. Vink M. (2010). Reduced functional coupling in the default-mode network during self-referential processing. *Hum. Brain Mapp.* 31 1117–1127. 10.1002/hbm.20920 20108218 PMC6870730

[B73] Veer I. M. Beckmann C. F. van Tol M. J. Ferrarini L. Milles J. Veltman D. J. (2010). Whole brain resting-state analysis reveals decreased functional connectivity in major depression. *Front. Syst. Neurosci.* 4:41. 10.3389/fnsys.2010.00041 20941370 PMC2950744

[B74] Verhallen A. M. Renken R. J. Marsman J. B. C. Ter Horst G. J. (2019). Romantic relationship breakup: an experimental model to study effects of stress on depression (-like) symptoms. *PLoS One* 14:e0217320. 10.1371/journal.pone.0217320 31150432 PMC6544239

[B75] Vohryzek J. Deco G. Cessac B. Kringelbach M. L. Cabral J. (2020). Ghost attractors in spontaneous brain activity: recurrent excursions into functionally-relevant BOLD phase-locking states. *Front. Syst. Neurosci.* 14:20. 10.3389/fnsys.2020.00020 32362815 PMC7182014

[B76] Wang L. Hermens D. F. Hickie I. B. Lagopoulos J. (2012). A systematic review of resting-state functional-MRI studies in major depression. *J. Affect. Disord.* 142 6–12. 10.1016/J.JAD.2012.04.013 22858266

[B77] Wei X. Shen H. Ren J. Li X. Xu X. Yang R. (2014). Altered resting-state connectivity in college students with nonclinical depressive symptoms. *PLoS One* 9:e114603. 10.1371/journal.pone.0114603 25502215 PMC4264752

[B78] Wise T. Marwood L. Perkins A. M. Herane-Vives A. Joules R. Lythgoe D. J. (2017). Instability of default mode network connectivity in major depression: a two-sample confirmation study. *Transl. Psychiatry* 7:e1105. 10.1038/tp.2017.40 28440813 PMC5416685

[B79] Wu H. Sun H. Xu J. Wu Y. Wang C. Xiao J. (2016). Changed hub and corresponding functional connectivity of subgenual anterior cingulate cortex in major depressive disorder. *Front. Neuroanat.* 10:120. 10.3389/fnana.2016.00120 28018183 PMC5159433

[B80] Yan C. G. Chen X. Li L. Castellanos F. X. Bai T. J. Bo Q. J. (2019). Reduced default mode network functional connectivity in patients with recurrent major depressive disorder. *Proc. Natl. Acad. Sci. U.S.A.* 116 9078–9083. 10.1073/pnas.1900390116 30979801 PMC6500168

[B81] Yeo B. T. T. Krienen F. M. Sepulcre J. Sabuncu M. R. Lashkari D. Hollinshead M. (2011). The organization of the human cerebral cortex estimated by intrinsic functional connectivity. *J. Neurophysiol.* 106 1125–1165. 10.1152/jn.00338.2011 21653723 PMC3174820

[B82] Zalesky A. Fornito A. Bullmore E. T. (2010). Network-based statistic: identifying differences in brain networks. *Neuroimage* 53 1197–1207. 10.1016/j.neuroimage.2010.06.041 20600983

[B83] Zalesky A. Fornito A. Cocchi L. Gollo L. L. Breakspear M. (2014). Time-resolved resting-state brain networks. *Proc. Natl. Acad. Sci. U.S.A.* 111 10341–10346. 10.1073/pnas.1400181111 24982140 PMC4104861

[B84] Zeng L.-L. Shen H. Liu L. Wang L. Li B. Fang P. (2012). Identifying major depression using whole-brain functional connectivity: a multivariate pattern analysis. *Brain* 135 1498–1507. 10.1093/brain/aws059 22418737

[B85] Zhi D. Calhoun V. D. Lv L. Ma X. Ke Q. Fu Z. (2018). Aberrant dynamic functional network connectivity and graph properties in major depressive disorder. *Front. Psychiatry* 9:339. 10.3389/fpsyt.2018.00339 30108526 PMC6080590

[B86] Zhu X. Wang X. Xiao J. Liao J. Zhong M. Wang W. (2012). Evidence of a dissociation pattern in resting-state default mode network connectivity in first-episode, treatment-naive major depression patients. *Biol. Psychiatry* 71 611–617. 10.1016/j.biopsych.2011.10.035 22177602

[B87] Zisook S. Simon N. M. Reynolds C. F. Pies R. Lebowitz B. Young I. T. (2010). Bereavement, complicated grief, and DSM, part 2: complicated grief. *J. Clin. Psychiatry* 71 1097–1098. 10.4088/JCP.10ac06391blu 20797383 PMC3754834

