# Supplementary material for: The Dynamics of Functional Brain Networks Associated With Depressive Symptoms in a Nonclinical Sample
Source: Front Neural Circuits. 2020 Sep 18;14:570583. doi: 10.3389/fncir.2020.570583 (PMC7530893; doi:10.3389/fncir.2020.570583)
Supplement: Supplementary file 1 [file Data_Sheet_1.PDF]

Supplementary tables

Supplementary Table S1. Demographic data of participants in the low and high MDI group

		Low MDI	High MDI	Between-group statistics		
		(n=50)	(n=19)	χ^2	t	p
Female	N (%)	21 (42%)	16 (84.2%)	9.86		0.002
Age (years)	Mean (SD)	22.1 (1.92)	22.21 (1.81)		-0.22	0.83
MDI	Mdn (Q1/Q3)	7 (5/12)	29 (24/33.7)		-14.19	<0.001
Time since breakup (months)	Mean (SD)	2.76 (1.51)	2.05 (1.52)		1.74	0.09
Relationship duration (months)	Mean (SD)	25.04 (17.79)	30.58 (16.40)		-1.18	0.24

MDI= Major Depression Inventory; χ^2= chi-square test statistic; p= p-value; t= independent-sample t-test statistic

Supplementary Table S2. Coordinates and abbreviations for the regions used in this study: 217 areas from Power et al. (2011) and 6 sub-cortical (in red).

x	y	z	label	net
-7	-52	61	Precuneus	SMT
-14	-18	40	Cingulate Gyrus	SMT
0	-15	47	Paracentral Lobule	SMT
10	-2	45	Cingulate Gyrus	SMT
-7	-21	65	Medial Frontal Gyrus	SMT
-54	-23	43	Postcentral Gyrus	SMT
-40	-19	54	Postcentral Gyrus	SMT
29	-39	59	Postcentral Gyrus	SMT
50	-20	42	Postcentral Gyrus	SMT
20	-29	60	Precentral Gyrus	SMT
44	-8	57	Precentral Gyrus	SMT
-29	-43	61	Postcentral Gyrus	SMT
22	-42	69	Postcentral Gyrus	SMT
-45	-32	47	Inferior Parietal Lobule	SMT
-21	-31	61	Postcentral Gyrus	SMT
42	-20	55	Postcentral Gyrus	SMT
2	-28	60	Medial Frontal Gyrus	SMT
3	-17	58	Medial Frontal Gyrus	SMT
38	-17	45	Precentral Gyrus	SMT
-49	-11	35	Precentral Gyrus	SMT
36	-9	14	Insula	SMT
51	-6	32	Precentral Gyrus	SMT
-53	-10	24	Precentral Gyrus	SMT

x	y	z	label	net
66	-8	25	Precentral Gyrus	SMT
47	-30	49	Postcentral Gyrus	SMT
-3	2	53	Medial Frontal Gyrus	CO
54	-28	34	Inferior Parietal Lobule	CO
19	-8	64	Middle Frontal Gyrus	CO
-10	-2	42	Cingulate Gyrus	CO
37	1	-4	Insula	CO
7	8	51	Medial Frontal Gyrus	CO
-45	0	9	Precentral Gyrus	CO
49	8	-1	Superior Temporal Gyrus	CO
-34	3	4	Claustrum	CO
-51	8	-2	Superior Temporal Gyrus	CO
-5	18	34	Cingulate Gyrus	CO
36	10	1	Insula	CO
32	-26	13	Insula	AUD
65	-33	20	Superior Temporal Gyrus	AUD
58	-16	7	Superior Temporal Gyrus	AUD
-38	-33	17	Insula	AUD
-60	-25	14	Superior Temporal Gyrus	AUD
-49	-26	5	Superior Temporal Gyrus	AUD
43	-23	20	Insula	AUD
-50	-34	26	Inferior Parietal Lobule	AUD
-53	-22	23	Postcentral Gyrus	AUD

-55	-9	12	Precentral Gyrus	AUD
56	-5	13	Precentral Gyrus	AUD
59	-17	29	Postcentral Gyrus	AUD
-30	-27	12	Insula	AUD
-41	-75	26	Middle Temporal Gyrus	DM
-13	-40	1	Parahippocampa Gyrus	DM
-46	-61	21	Middle Temporal Gyrus	DM
43	-72	28	Middle Temporal Gyrus	DM
-44	-65	35	Angular Gyrus	DM
-7	-55	27	Cingulate Gyrus	DM
6	-59	35	Precuneus	DM
-11	-56	16	Posterior Cingulate	DM
-3	-49	13	Posterior Cingulate	DM
8	-48	31	Cingulate Gyrus	DM
15	-63	26	Precuneus	DM
-2	-37	44	Cingulate Gyrus	DM
11	-54	17	Posterior Cingulate	DM
52	-59	36	Angular Gyrus	DM
23	33	48	Middle Frontal Gyrus	DM
-10	39	52	Superior Frontal Gyrus	DM
-16	29	53	Superior Frontal Gyrus	DM
-35	20	51	Middle Frontal Gyrus	DM
22	39	39	Superior Frontal Gyrus	DM
-20	45	39	Superior Frontal Gyrus	DM
6	54	16	Medial Frontal Gyrus	DM
6	64	22	Medial Frontal Gyrus	DM
-7	51	-1	Medial Frontal Gyrus	DM
9	54	3	Medial Frontal Gyrus	DM
8	42	-5	Medial Frontal Gyrus	DM
-11	45	8	Medial Frontal Gyrus	DM
-2	38	36	Medial Frontal Gyrus	DM
-3	42	16	Anterior Cingulate	DM
-20	64	19	Superior Frontal Gyrus	DM
-8	48	23	Medial Frontal Gyrus	DM
65	-12	-19	Inferior Temporal Gyrus	DM
-56	-13	-10	Middle Temporal Gyrus	DM
-58	-30	-4	Middle Temporal Gyrus	DM
65	-31	-9	Middle Temporal Gyrus	DM
13	30	59	Superior Frontal Gyrus	DM
12	36	20	Anterior Cingulate	DM
52	-2	-16	Middle Temporal Gyrus	DM
-26	-40	-8	Parahippocampa Gyrus	DM
27	-37	-13	Parahippocampa Gyrus	DM
-34	-38	-16	Fusiform Gyrus	DM
-53	3	-27	Middle Temporal Gyrus	DM
47	-50	29	Supramarginal Gyrus	DM
-49	-42	1	Middle Temporal Gyrus	DM
-46	31	-13	Inferior Frontal Gyrus	DM
49	35	-12	Inferior Frontal Gyrus	DM
-2	-35	31	Cingulate Gyrus	MR
-7	-71	42	Precuneus	MR
11	-66	42	Precuneus	MR
4	-48	51	Precuneus	MR
2	-24	30	Cingulate Gyrus	MR
18	-47	-10	Parahippocampa Gyrus	VIS
40	-72	14	Middle Temporal Gyrus	VIS
8	-72	11	Cuneus	VIS
-8	-81	7	Cuneus	VIS
-28	-79	19	Sub-Gyral	VIS
20	-66	2	Lingual Gyrus	VIS
-24	-91	19	Cuneus	VIS
27	-59	-9	Lingual Gyrus	VIS
-15	-72	-8	Lingual Gyrus	VIS
-18	-68	5	Cuneus	VIS
43	-78	-12	Inferior Occipital Gyrus	VIS
-47	-76	-10	Middle Occipital Gyrus	VIS
29	-77	25	Sub-Gyral	VIS
20	-86	-2	Lingual Gyrus	VIS
15	-77	31	Cuneus	VIS
-16	-52	-1	Lingual Gyrus	VIS
42	-66	-8	Middle Occipital Gyrus	VIS
24	-87	24	Cuneus	VIS
6	-72	24	Precuneus	VIS
-42	-74	0	Middle Occipital Gyrus	VIS
26	-79	-16	Lingual Gyrus	VIS
-16	-77	34	Precuneus	VIS
-3	-81	21	Cuneus	VIS
-40	-88	-6	Inferior Occipital Gyrus	VIS
37	-84	13	Middle Occipital Gyrus	VIS
6	-81	6	Cuneus	VIS
-26	-90	3	Middle Occipital Gyrus	VIS
-33	-79	-13	Middle Occipital Gyrus	VIS
37	-81	1	Middle Occipital Gyrus	VIS
-44	2	46	Middle Frontal Gyrus	FP
48	25	27	Middle Frontal Gyrus	FP
-47	11	23	Inferior Frontal Gyrus	FP
-53	-49	43	Inferior Parietal Lobule	FP
-23	11	64	Superior Frontal Gyrus	FP
58	-53	-14	Middle Temporal Gyrus	FP
47	10	33	Middle Frontal Gyrus	FP
-41	6	33	Inferior Frontal Gyrus	FP
-42	38	21	Middle Frontal Gyrus	FP
38	43	15	Middle Frontal Gyrus	FP
49	-42	45	Inferior Parietal Lobule	FP
-28	-58	48	Superior Parietal Lobule	FP
44	-53	47	Inferior Parietal Lobule	FP

32	14	56	Superior Frontal Gyrus	FP
37	-65	40	Inferior Parietal Lobule	FP
-42	-55	45	Inferior Parietal Lobule	FP
40	18	40	Middle Frontal Gyrus	FP
-34	55	4	Middle Frontal Gyrus	FP
-42	45	-2	Middle Frontal Gyrus	FP
33	-53	44	Inferior Parietal Lobule	FP
43	49	-2	Middle Frontal Gyrus	FP
-42	25	30	Middle Frontal Gyrus	FP
-3	26	44	Medial Frontal Gyrus	FP
11	-39	50	Paracentral Lobule	SAL
55	-45	37	Supramarginal Gyrus	SAL
42	0	47	Middle Frontal Gyrus	SAL
31	33	26	Sub-Gyral	SAL
48	22	10	Inferior Frontal Gyrus	SAL
-35	20	0	Extra-Nuclear	SAL
36	22	3	Insula	SAL
37	32	-2	Inferior Frontal Gyrus	SAL
34	16	-8	Extra-Nuclear	SAL
-11	26	25	Anterior Cingulate	SAL
-1	15	44	Cingulate Gyrus	SAL
-28	52	21	Middle Frontal Gyrus	SAL
0	30	27	undefined	SAL
5	23	37	Cingulate Gyrus	SAL
10	22	27	Anterior Cingulate	SAL
31	56	14	Middle Frontal Gyrus	SAL
26	50	27	Superior Frontal Gyrus	SAL
-39	51	17	Superior Frontal Gyrus	SAL
6	-24	0	Extra-Nuclear	SC
-2	-13	12	Extra-Nuclear	SC
-10	-18	7	Thalamus	SC
12	-17	8	Thalamus	SC
-5	-28	-4	undefined	SC
-22	7	-5	Lentiform Nucleus	SC
-15	4	8	Lentiform Nucleus	SC
31	-14	2	Extra-Nuclear	SC
23	10	1	Lentiform Nucleus	SC
29	1	4	Extra-Nuclear	SC
-31	-11	0	Extra-Nuclear	SC
15	5	7	Extra-Nuclear	SC
9	-4	6	Thalamus	SC
-22	-6	-16	Amygdala	SC
24	-2	-18	Amygdala	SC
-10	14	4	Caudate	SC
12	16	4	Caudate	SC
-26	-16	-20	Hippocampus	SC
26	-14	-20	Hippocampus	SC
54	-43	22	Inferior Parietal Lobule	VAT
-56	-50	10	Superior Temporal Gyrus	VAT
-55	-40	14	Superior Temporal Gyrus	VAT
52	-33	8	Superior Temporal Gyrus	VAT
51	-29	-4	Middle Temporal Gyrus	VAT
56	-46	11	Superior Temporal Gyrus	VAT
53	33	1	Inferior Frontal Gyrus	VAT
-49	25	-1	Inferior Frontal Gyrus	VAT
10	-62	61	Precuneus	DAT
-52	-63	5	Middle Temporal Gyrus	DAT
22	-65	48	Precuneus	DAT
46	-59	4	Middle Temporal Gyrus	DAT
25	-58	60	Superior Parietal Lobule	DAT
-33	-46	47	Sub-Gyral	DAT
-27	-71	37	Precuneus	DAT
-32	-1	54	Middle Frontal Gyrus	DAT
-42	-60	-9	Sub-Gyral	DAT
-17	-59	64	Superior Parietal Lobule	DAT
29	-5	54	Middle Frontal Gyrus	DAT
-16	-65	-20	Declive	Cereb
-32	-55	-25	Culmen	Cereb
22	-58	-23	Declive	Cereb
1	-62	-18	Declive	Cereb
-25	-98	-12	Lingual Gyrus	U
-21	-22	-20	Parahippocampa Gyrus	U
17	-28	-17	Culmen	U
34	38	-12	Middle Frontal Gyrus	U
27	16	-17	Inferior Frontal Gyrus	U
-31	19	-19	Inferior Frontal Gyrus	U
8	-91	-7	Lingual Gyrus	U
-12	-95	-13	Lingual Gyrus	U
-18	-76	-24	Declive	U
-47	-51	-21	Fusiform Gyrus	U
46	-47	-17	Fusiform Gyrus	U

SMT = somatosensory/motor; CO = cingulo-opercular task control; AUD = Auditory; DM = default mode; MR = memory retrieval; VIS = Visual; FP = frontoparietal task control; SAL = salience; SC = subcortical; VAT = ventral attentional; DAT = dorsal attentional; Cereb = cerebellar; U = uncertain.

Supplementary Table S3. Pairwise connections between two nodes within the significant network differences between low and high MDI.

Node 1		Node 2		t
Precuneus.R	DM	Insula.L	AUD	3.53
		Precentral Gyrus.L	AUD	3.64
		Medial Frontal Gyrus.L	SMT	4.03
		Medial Frontal Gyrus.R	SMT	4.58
		Medial Frontal Gyrus.R	SMT	4.54
		Lingual Gyrus.R	VIS	4.19
		Inferior Occipital Gyrus.R	VIS	3.67
		Superior Temporal Gyrus.L	VAT	4.2
		Superior Temporal Gyrus.R	VAT	3.88
Posterior Cingulate.R	DM	Precentral Gyrus.L	AUD	3.74
		Medial Frontal Gyrus.R	SMT	3.65
		Medial Frontal Gyrus.R	SMT	3.6
		Precentral Gyrus.R	SMT	3.71
		Parahippocampa Gyrus.R	VIS	3.53
		Middle Occipital Gyrus.R	VIS	3.54
		Superior Temporal Gyrus.R	VAT	3.71
Middle Temporal Gyrus.L	DM	Medial Frontal Gyrus.L	CO	3.72
		Medial Frontal Gyrus.R	CO	4.01
		Superior Temporal Gyrus.R	CO	4.04
		Middle Frontal Gyrus.R	DAT	3.87
		Middle Frontal Gyrus.R	SAL	3.53
Medial Frontal Gyrus.R	DM	Superior Temporal Gyrus.L	AUD	3.7
		Middle Frontal Gyrus.R	CO	3.78
		Superior Temporal Gyrus.R	CO	4
		Middle Frontal Gyrus.R	DAT	3.98
Middle Temporal Gyrus.L	DM	Precentral Gyrus.R	SMT	3.77
		Inferior Parietal Lobule.R	FPN	3.84
		Inferior Parietal Lobule.R	VAT	4.16
Middle Temporal Gyrus.R	DM	Insula.R	CO	3.79
		Superior Temporal Gyrus.R	CO	3.86
Posterior Cingulate.L	DM	Precentral Gyrus.L	AUD	3.79
		Postcentral Gyrus.R	SMT	3.66
Middle Temporal Gyrus.R	DM	Insula.R	CO	3.55
Superior Frontal Gyrus.L	DM	Middle Frontal Gyrus.R	DAT	5
Superior Temporal Gyrus.L	VAT	Middle Frontal Gyrus.R	DAT	3.52
		Precentral Gyrus.R	SMT	4.04
Superior Temporal Gyrus.L	VAT	Precentral Gyrus.R	SMT	4.34
Lingual Gyrus.R	VIS	Paracentral Lobule.L	SMT	3.53
Precuneus.L	VIS	Precentral Gyrus.R	SMT	3.98
Precuneus.R	VIS	Medial Frontal Gyrus.R	SMT	4.05
Superior Temporal Gyrus.R	AUD	Superior Temporal Gyrus.L	VAT	4.1
Middle Frontal Gyrus.R	CO	Paracentral Lobule.L	SMT	3.67

t: *t*-test statistical values for the functional connectivity differences between high and low MDI; L: left; M: right; AUD: auditory; CO: cingulo-opercular task control; DM: default mode network; DAT: dorsal attention network; FPN: frontoparietal network; SAL: salience network; SMT: somatosensory motor; VAT: ventral attention network; VIS: visual

Supplementary Table S4. Repertoire of recurrent phase-locking (PL) states obtained by clustering the leading eigenvectors into 9 clusters.

		**Low MDI**	**High MDI**		
		Mean ± SD	Mean ± SD	p	p-FDR
Occupancy					
	PL state 1	20 ± 12.4%	17 ± 13.2%	0.232	0.299
	PL state 2	12.5 ± 8%	9.5 ± 6.5%	0.177	0.265
	PL state 3	9.8 ± 8%	16.1 ± 8.8%	0.003	**0.012**
	PL state 4	10.6 ± 5.5%	10.5 ± 7%	0.413	0.413
	PL state 5	8.2 ± 6.9%	13.8 ± 11.9%	0.047	0.109
	PL state 6	8.3 ± 6.6%	13 ± 9.9%	0.062	0.112
	PL state 7	10.9 ± 8.1%	5.3 ± 7.3%	0.049	0.109
	PL state 8	10.6 ± 6.6%	6.2 ± 3.4%	0.001	**0.004**
	PL state 9	9.1 ± 6.9%	8.8 ± 5.8%	0.394	0.413
Lifetime					
	PL state 1	7.35 ± 3.45s	6.83 ± 3.09s	0.193	0.345
	PL state 2	5.57 ± 2.13s	5.13 ± 1.93s	0.374	0.455
	PL state 3	5.04 ± 2.60s	6.8 ± 2.88s	0.010	**0.041**
	PL state 4	4.81 ± 1.55s	4.51 ± 1.96s	0.230	0.345
	PL state 5	4.8 ± 2.09s	6.15 ± 3s	0.079	0.178
	PL state 6	4.53 ± 2.02s	4.84 ± 2.55s	0.484	0.484
	PL state 7	5.77 ± 2.57s	3.91 ± 1.72s	0.008	**0.041**
	PL state 8	5.54 ± 2.25s	4.05 ± 1.97s	0.014	**0.041**
	PL state 9	5.13 ± 2.25s	5.46 ± 3.09s	0.405	0.455

Analysis via nonparametric permutation-based t-test (N=69 participants); p-value (alpha=0.05); p-FDR= false discovery rate adjusted p-value; SD= standard deviation.
