# Supplementary material for: The Dynamics of Functional Brain Networks Associated With Depressive Symptoms in a Nonclinical Sample
Source: Front Neural Circuits. 2020 Sep 18;14:570583. doi: 10.3389/fncir.2020.570583 (PMC7530893; doi:10.3389/fncir.2020.570583)
Supplement: Supplementary file 2 [file Image_1.PDF]

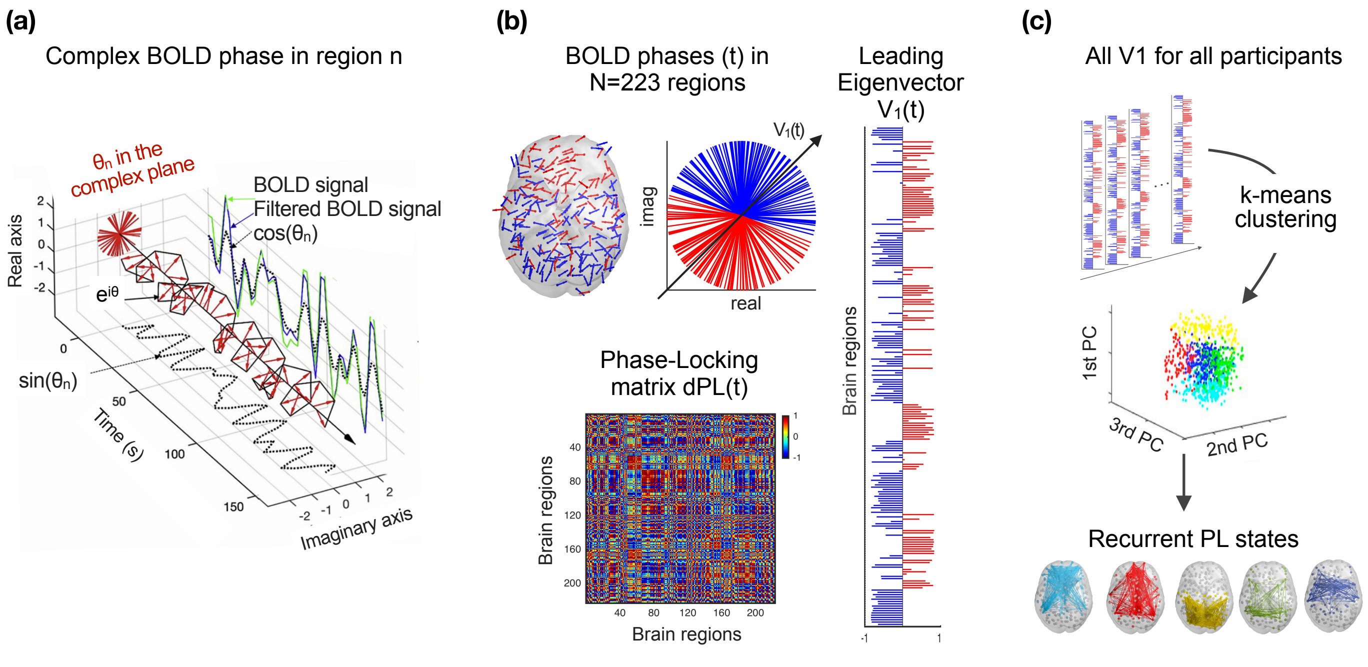
(a)
Complex BOLD phase in region n
θn in the complex plane
BOLD signal
Filtered BOLD signal
cos(θn)
Real axis
e^iθ
sin(θn)
Time (s)
Imaginary axis
(b)
BOLD phases (t) in N=223 regions
V1(t)
imag
real
Leading Eigenvector V1(t)
Phase-Locking matrix dPL(t)
Brain regions
Brain regions
Brain regions
(c)
All V1 for all participants
k-means clustering
1st PC
3rd PC
2nd PC
Recurrent PL states
