# Supplementary material for: The Dynamics of Functional Brain Networks Associated With Depressive Symptoms in a Nonclinical Sample
Source: Front Neural Circuits. 2020 Sep 18;14:570583. doi: 10.3389/fncir.2020.570583 (PMC7530893; doi:10.3389/fncir.2020.570583)
Supplement: Supplementary file 3 [file Image_2.PDF]

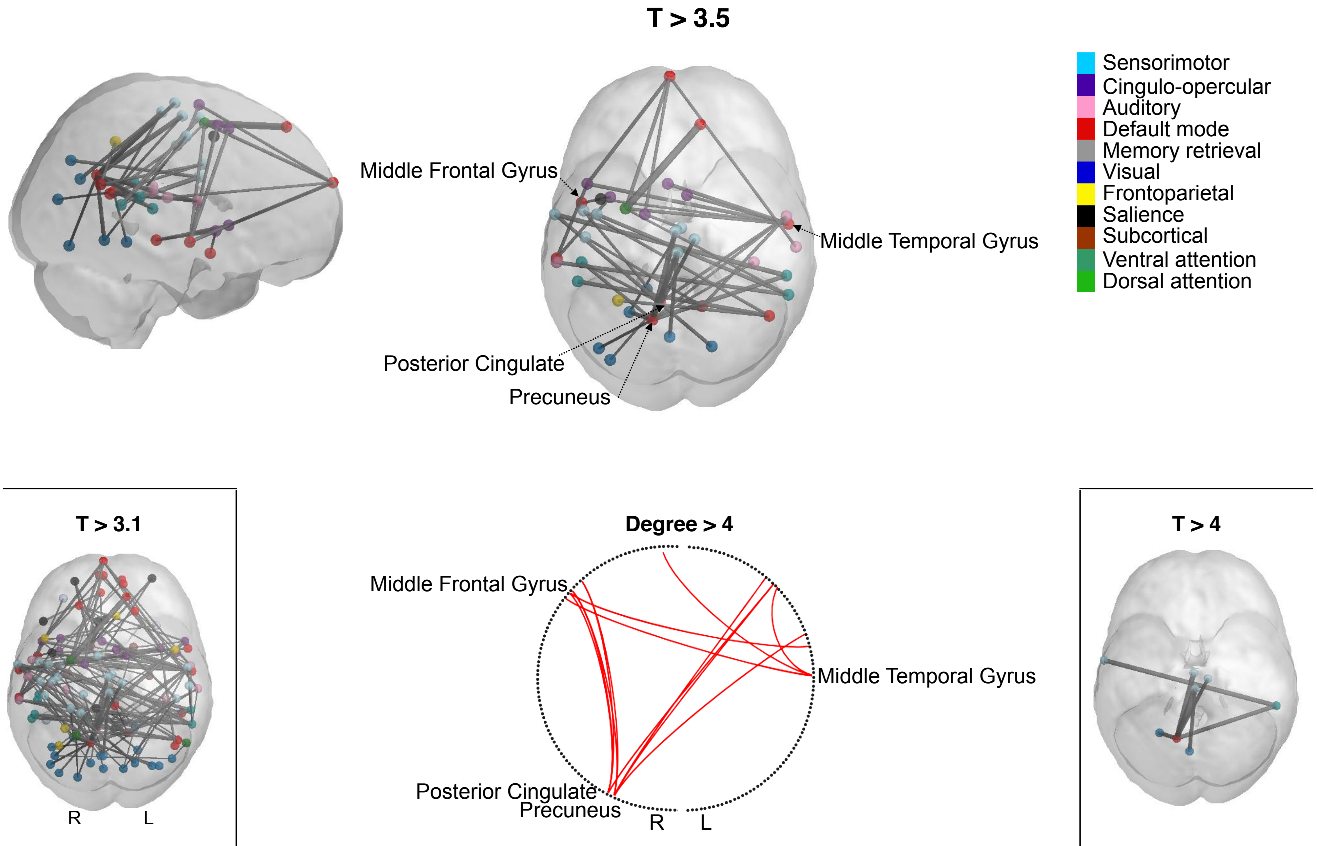
T > 3.5
Sensorimotor
Cingulo-opercular
Auditory
Default mode
Memory retrieval
Visual
Frontoparietal
Salience
Subcortical
Ventral attention
Dorsal attention
Middle Frontal Gyrus
Middle Temporal Gyrus
Posterior Cingulate
Precuneus
T > 3.1
R
L
Degree > 4
Middle Frontal Gyrus
Middle Temporal Gyrus
Posterior Cingulate
Precuneus
R
L
T > 4
