# Supplementary figures and images for: The Dynamics of Functional Brain Networks Associated With Depressive Symptoms in a Nonclinical Sample
Source: Front Neural Circuits. 2020 Sep 18;14:570583. doi: 10.3389/fncir.2020.570583 (PMC7530893; doi:10.3389/fncir.2020.570583)

**(a)** Significant PL states in each partition model

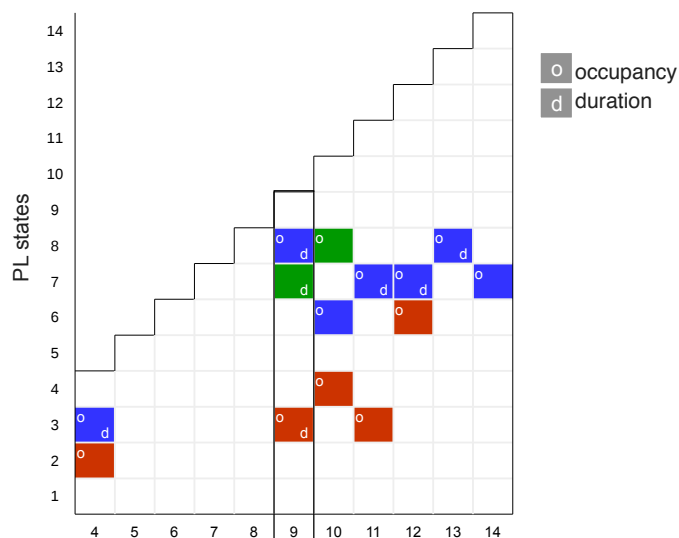

**(b)**

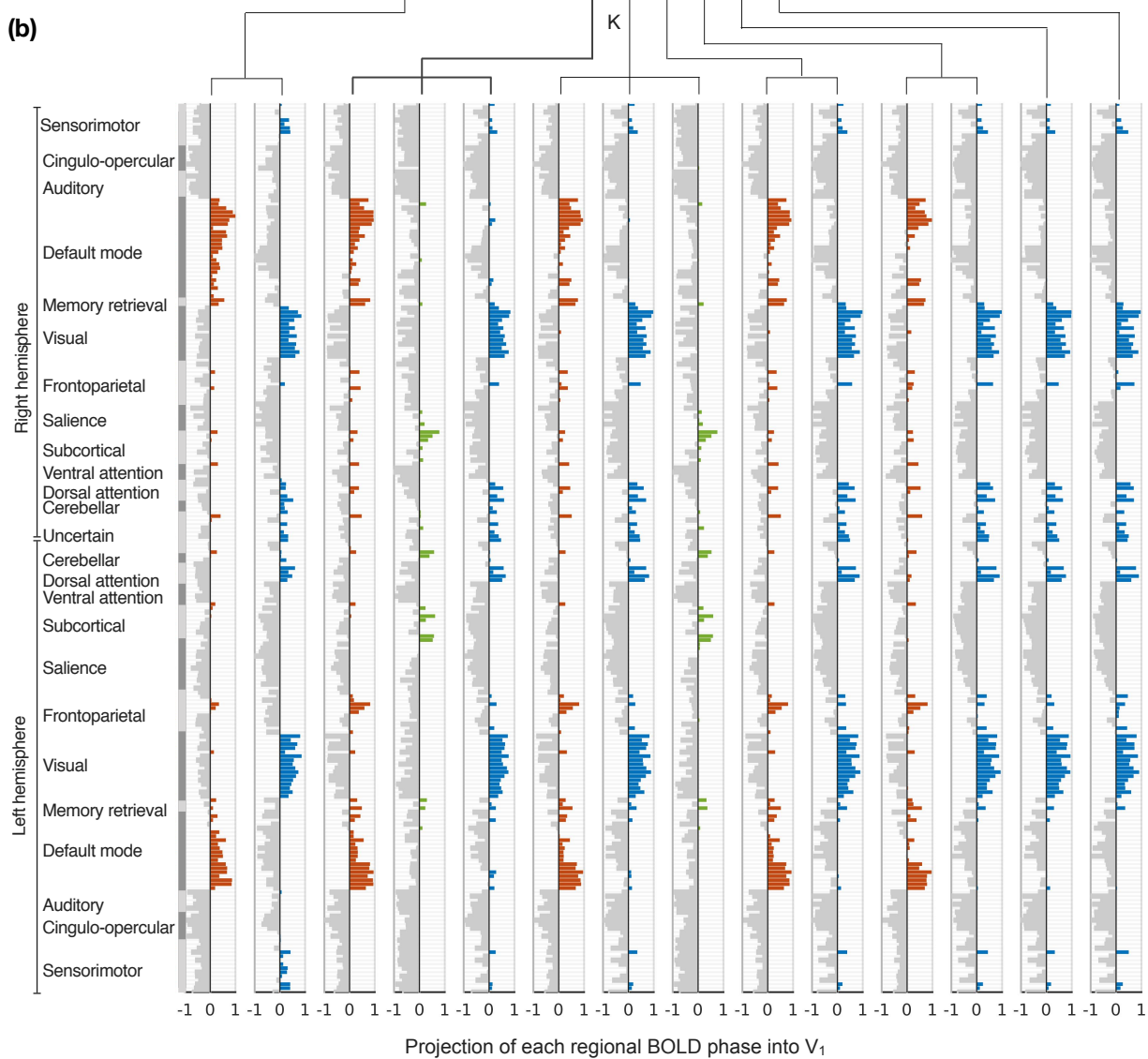

Supplement: Supplementary file 4 [file Image_3.PDF]

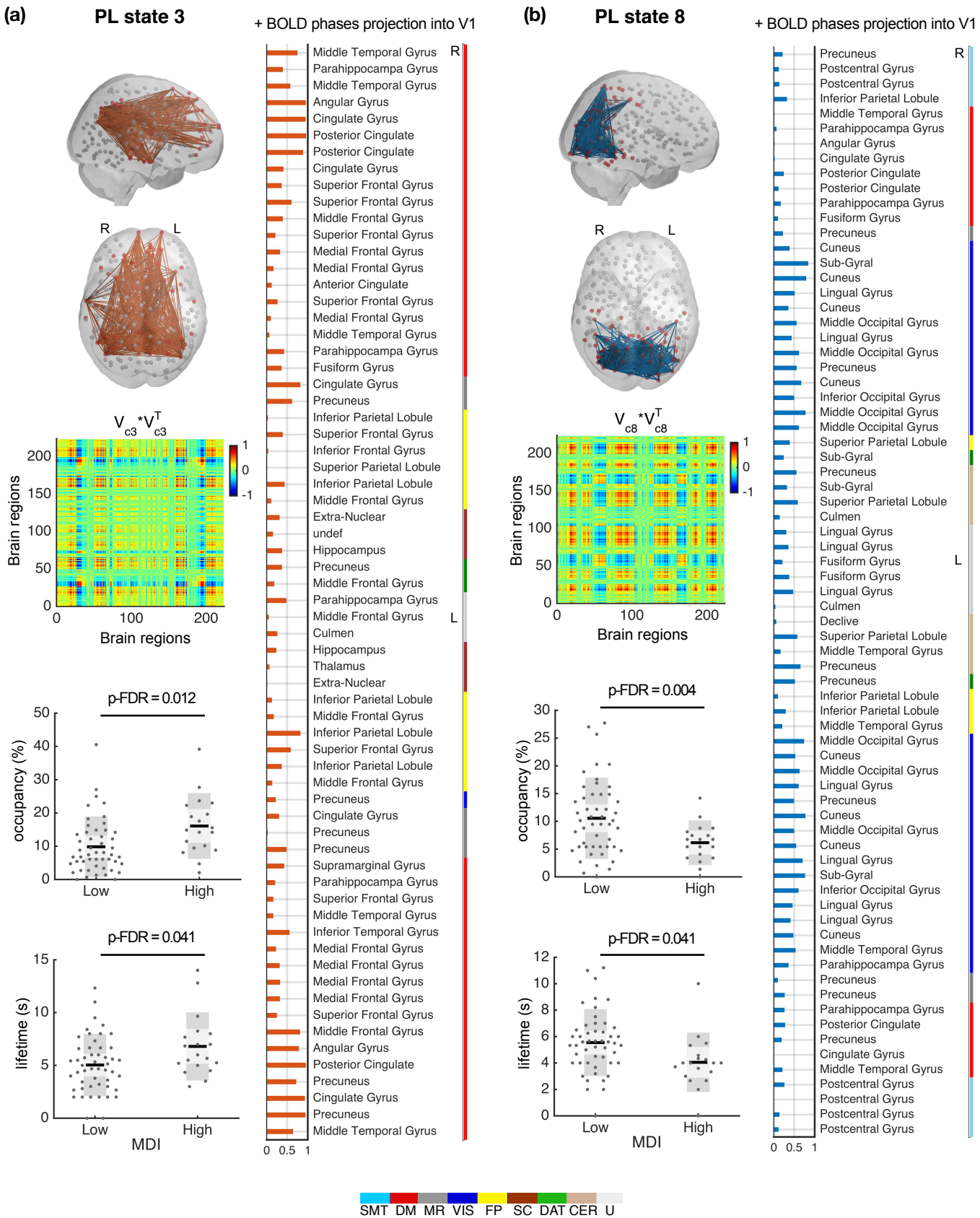

Supplement: Supplementary file 5 [file Image_4.PDF]
