# Supplementary material for: The Dynamics of Functional Brain Networks Associated With Depressive Symptoms in a Nonclinical Sample
Source: Front Neural Circuits. 2020 Sep 18;14:570583. doi: 10.3389/fncir.2020.570583 (PMC7530893; doi:10.3389/fncir.2020.570583)
Supplement: Supplementary file 6 [file Image_5.PDF]

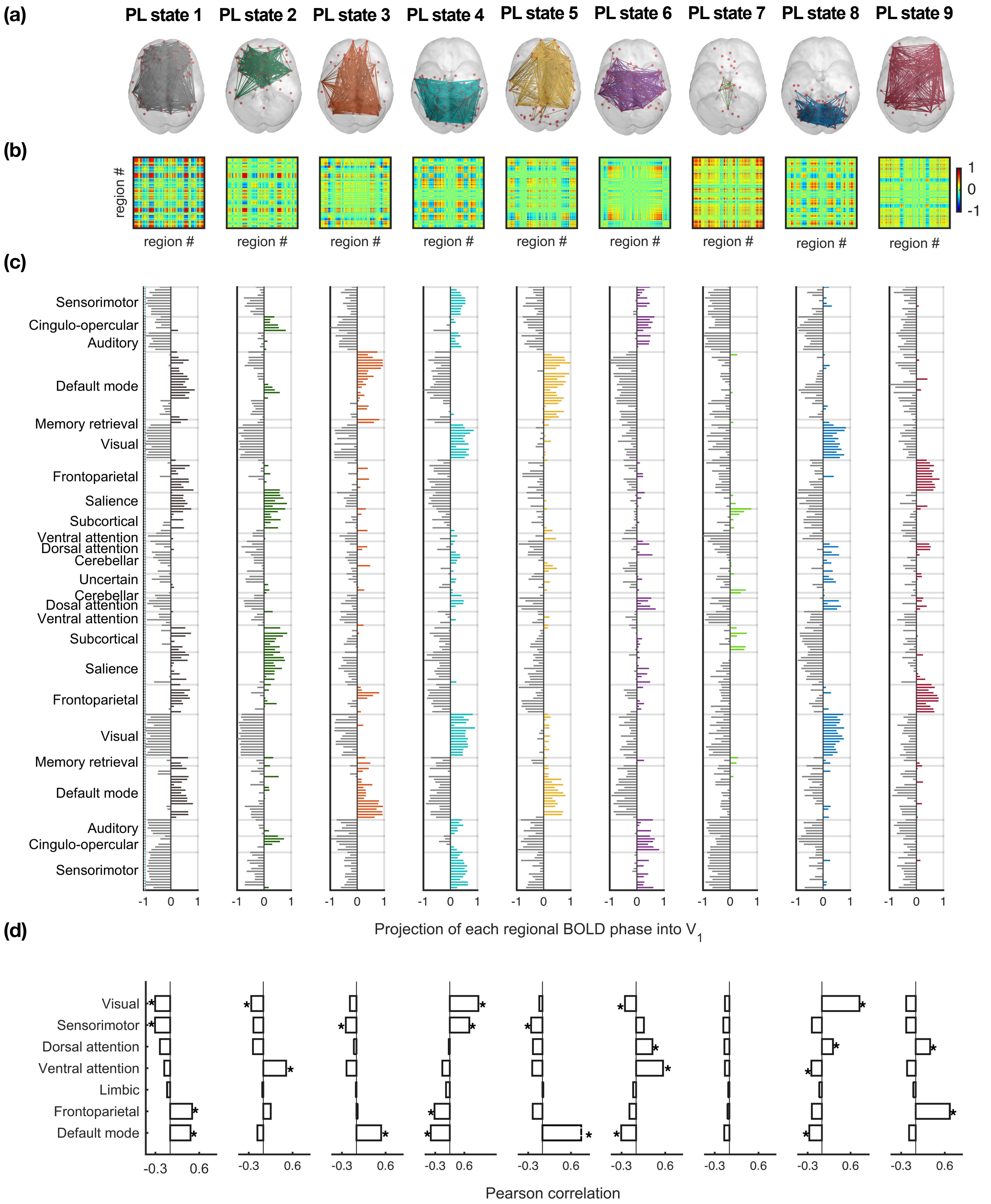
(a)
PL state 1
PL state 2
PL state 3
PL state 4
PL state 5
PL state 6
PL state 7
PL state 8
PL state 9
(b)
region #
region #
1
0
-1
(c)
Sensorimotor
Cingulo-opercular
Auditory
Default mode
Memory retrieval
Visual
Frontoparietal
Salience
Subcortical
Ventral attention
Dorsal attention
Cerebellar
Uncertain
Cerebellar
Dosal attention
Ventral attention
Subcortical
Salience
Frontoparietal
Visual
Memory retrieval
Default mode
Auditory
Cingulo-opercular
Sensorimotor
-1 0 1
Projection of each regional BOLD phase into V1
(d)
Visual
Sensorimotor
Dorsal attention
Ventral attention
Limbic
Frontoparietal
Default mode
-0.3 0.6
Pearson correlation
