# Supplementary material for: The Dynamics of Functional Brain Networks Associated With Depressive Symptoms in a Nonclinical Sample
Source: Front Neural Circuits. 2020 Sep 18;14:570583. doi: 10.3389/fncir.2020.570583 (PMC7530893; doi:10.3389/fncir.2020.570583)
Supplement: Supplementary file 7 [file Image_6.PDF]

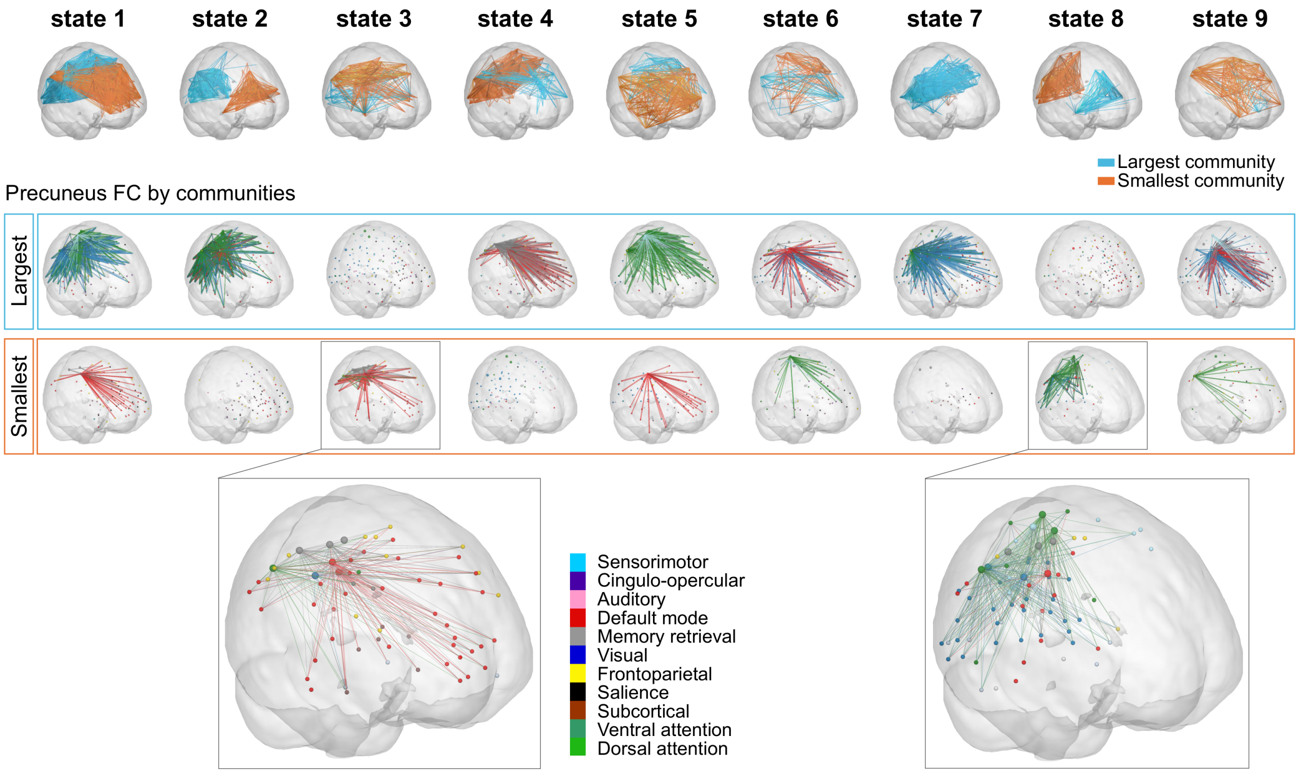
state 1
state 2
state 3
state 4
state 5
state 6
state 7
state 8
state 9
Largest community
Smallest community
Precuneus FC by communities
Largest
Smallest
Sensorimotor
Cingulo-opercular
Auditory
Default mode
Memory retrieval
Visual
Frontoparietal
Salience
Subcortical
Ventral attention
Dorsal attention
