# Supplementary material for: The Dynamics of Functional Brain Networks Associated With Depressive Symptoms in a Nonclinical Sample
Source: Front Neural Circuits. 2020 Sep 18;14:570583. doi: 10.3389/fncir.2020.570583 (PMC7530893; doi:10.3389/fncir.2020.570583)
Supplement: Supplementary file 8 [file Image_7.PDF]

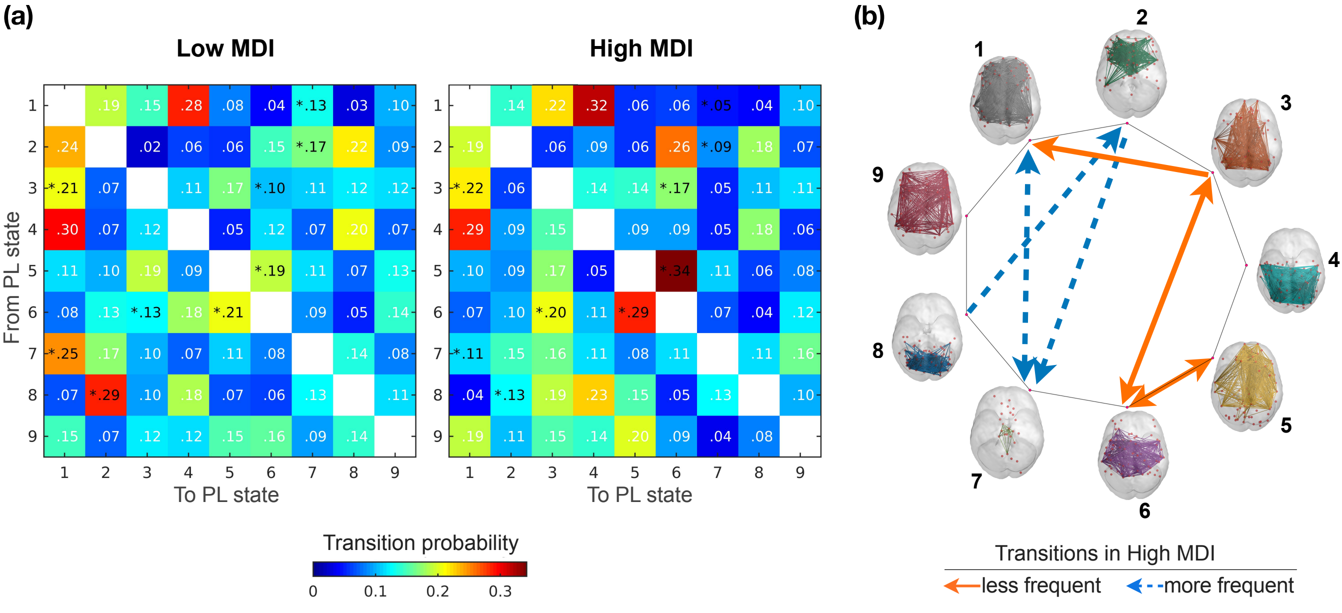
(a)
Low MDI
High MDI
From PL state
To PL state
To PL state
1 .19 .15 .28 .08 .04 *.13 .03 .10
2 .24 .02 .06 .06 .15 *.17 .22 .09
3 *.21 .07 .11 .17 *.10 .11 .12 .12
4 .30 .07 .12 .05 .12 .07 .20 .07
5 .11 .10 .19 .09 *.19 .11 .07 .13
6 .08 .13 *.13 .18 *.21 .09 .05 .14
7 *.25 .17 .10 .07 .11 .08 .14 .08
8 .07 *.29 .10 .18 .07 .06 .13 .11
9 .15 .07 .12 .12 .15 .16 .09 .14
1 2 3 4 5 6 7 8 9
1 .14 .22 .32 .06 .06 *.05 .04 .10
2 .19 .06 .09 .06 .26 *.09 .18 .07
3 *.22 .06 .14 .14 *.17 .05 .11 .11
4 .29 .09 .15 .09 .09 .05 .18 .06
5 .10 .09 .17 .05 *.34 .11 .06 .08
6 .07 .10 *.20 .11 *.29 .07 .04 .12
7 *.11 .15 .16 .11 .08 .11 .11 .16
8 .04 *.13 .19 .23 .15 .05 .13 .10
9 .19 .11 .15 .14 .20 .09 .04 .08
1 2 3 4 5 6 7 8 9
Transition probability
0 0.1 0.2 0.3
(b)
1 2 3 4 5 6 7 8 9
Transitions in High MDI
less frequent
more frequent
